# Chemical respiratory sensitization—Current status of mechanistic understanding, knowledge gaps and possible identification methods of sensitizers

**DOI:** 10.3389/ftox.2024.1331803

**Published:** 2024-07-29

**Authors:** Rita Hargitai, Lucia Parráková, Tünde Szatmári, Pablo Monfort-Lanzas, Valentina Galbiati, Karine Audouze, Florence Jornod, Yvonne C. M. Staal, Sabina Burla, Aline Chary, Arno C. Gutleb, Katalin Lumniczky, Rob J. Vandebriel, Johanna M. Gostner

**Affiliations:** ^1^ Unit of Radiation Medicine, Department of Radiobiology and Radiohygiene, National Centre for Public Health and Pharmacy (NCPHP), Budapest, Hungary; ^2^ Biochemical Immunotoxicology Group, Institute of Medical Biochemistry, Medical University of Innsbruck (MUI), Innsbruck, Austria; ^3^ Institute of Bioinformatics, Medical University of Innsbruck (MUI), Innsbruck, Austria; ^4^ Laboratory of Toxicology, Department of Pharmacological and Biomolecular Sciences “Rodolfo Paoletti”, Università Degli Studi di Milano (UNIMI), Milano, Italy; ^5^ Inserm UMR-S 1124, Université Paris Cité, Paris, France; ^6^ Centre for Health Protection, National Institute of Public Health and the Environment (RIVM), Bilthoven, Netherlands; ^7^ Luxembourg Institute of Science and Technology (LIST), Belvaux, Luxembourg

**Keywords:** adverse outcome pathway (AOP), chemical respiratory allergy, chemical sensitizer, chemical-induced hypersensitivity, key event, new approach methodology (NAM), occupational asthma, respiratory sensitization

## Abstract

Respiratory sensitization is a complex immunological process eventually leading to hypersensitivity following re-exposure to the chemical. A frequent consequence is occupational asthma, which may occur after long latency periods. Although chemical-induced respiratory hypersensitivity has been known for decades, there are currently no comprehensive and validated approaches available for the prospective identification of chemicals that induce respiratory sensitization, while the expectations of new approach methodologies (NAMs) are high. A great hope is that due to a better understanding of the molecular key events, new methods can be developed now. However, this is a big challenge due to the different chemical classes to which respiratory sensitizers belong, as well as because of the complexity of the response and the late manifestation of symptoms. In this review article, the current information on respiratory sensitization related processes is summarized by introducing it in the available adverse outcome pathway (AOP) concept. Potentially useful models for prediction are discussed. Knowledge gaps and gaps of regulatory concern are identified.

## 1 Introduction

Chemical respiratory sensitization manifests clinically mostly as occupational asthma (OA), but also as rhinitis, conjunctivitis, and rarely as alveolitis (Cochrane et al., 2015; Seed et al., 2015; Golden et al., 2021). It is meanwhile recognized that occupational and environmental exposures can play a role in the development of almost all serious respiratory diseases in the general population (Feary et al., 2023). Even chronic low-level exposure may lead to long-term health effects. However, the link between asthma and chemical exposure is based on the patients medical history, and it is often difficult to trace the history of exposure and to identify the responsible chemicals (Vincent et al., 2017). Likewise, understanding the effects of respiratory sensitizer exposure on human health is limited due to difficulties in evaluating (occupational) exposure (Weisel, 2002; Batterman et al., 2014).

Asthma was officially recognized as the most prevalent occupational disease of the lungs in developed countries (Torén and Blanc, 2009) and is still the second-most common occupational lung disease after pneumoconiosis in developing countries (Üzmezoğlu, 2021). OA represents about 15% of all adult asthma cases (Vincent et al., 2017), and sensitizer-induced OA is the most common type of OA, being induced by either high-molecular-weight (HMW) or low-molecular-weight (LMW) agents (Feary et al., 2023). An estimated 10%–25% of adult asthma cases are associated with exposure to sensitizing or irritant agents with severe consequence for those affected, as for respiratory (immune-mediated) asthma mostly cessation of exposure is required and even then, complete recovery is not guaranteed (Kuruvilla et al., 2019a). As most part of the literature does not usually make a clear distinction between respiratory reactions triggered by sensitization of the respiratory tract and those triggered by other factors, it is not possible to provide precise information on the frequency. Concurrent exposure to particles that induce inflammation or irritation can aggravate the symptoms (Staal et al., 2014). Inflammation is a key factor, and patients with severe asthma, encompassing up to 10% of all asthma patients (Kuruvilla et al., 2019b), require anti-inflammatory corticosteroids as treatment.

The definition of work-related asthma includes immunologic OA, characterized by a latency period before the onset of symptoms, non-immunologic OA, which occurs after single or multiple exposures to high concentrations of irritant materials, work-aggravated asthma, which is preexisting, or concurrent asthma exacerbated by workplace exposures, as well as variant syndromes (Mapp et al., 2005). Chemicals can induce asthma via both immunological and non-immunological (irritant-induced) mechanisms, but this is usually less discussed and distinguishing the mechanisms is sometimes problematic (Pemberton and Kimber, 2021). According to the ECHA guidance on interpretation of the classification, labelling and packaging of substances and mixtures (CLP) criteria there is no need for the demonstration of an immunological mechanism to classify a chemical as respiratory sensitizer.

As mentioned above, occupational settings are an important source of exposure, but also in the general environment we are continuously exposed to inhalable substances, e.g., from paint and coatings, but also from medicines, electronics, cosmetics and consumer care products, etc., though it is not clear to which extent this increases the number of affected people due to diagnostic difficulties. For proteins (food and feed, pollen, animal dander, skin and sputum, invertebrates such as house dust mites), the pathophysiological mechanism of the allergic reaction that is based on IgE-induced mast cell degranulation upon inhalation is relatively well understood. However, the underlying mechanisms differ in several aspects from chemical-induced allergy for which the formation of haptens by binding to cellular macromolecules is a prerequisite to become immunogenic.

Inhalation exposure to chemicals represents the most common exposure route of concern for the induction of sensitization, but under some circumstances skin exposure may be effective for the acquisition of respiratory sensitization (Botham et al., 1988; Blaikie et al., 1995; Pauluhn et al., 2005; Tsui et al., 2020). The nature of the adaptive response is controlled by different T cell subpopulations. Overall, respiratory sensitizers preferentially elicit a T helper cell type 2 (Th2) response, while skin sensitizers promote Th1 type reactions. However, the effects of sensitizers are not limited to the airways. For example, haptenized self-proteins can contribute to the development of autoimmune diseases (Erkes and Selvan et al., 2014; Sakamoto et al., 2023).

Having entered into force in 2007, the regulation on the registration, evaluation, authorization and restriction of chemicals (REACH) is the main regulation for chemical risk assessment in the European Union that enforces the better identification of the intrinsic properties of chemicals (EC 1907/2006). Article 57 of the EU REACH regulation states that respiratory sensitizers are considered as substances of very high concern (SVHC). The current OECD testing methods for inhalable substances (no. 403: Acute Inhalation Toxicity, no. 412: Subacute Inhalation Toxicity: 28-Day Study, no. 413: Subchronic Inhalation Toxicity: 90-Day Study, no. 436: Acute Inhalation Toxicity – Acute Toxic Class Method and no. 433: Acute Inhalation Toxicity: Fixed Concentration Procedure) are still based on animal experiments, mainly utilizing rodents^1^.

**FIGURE 1:**
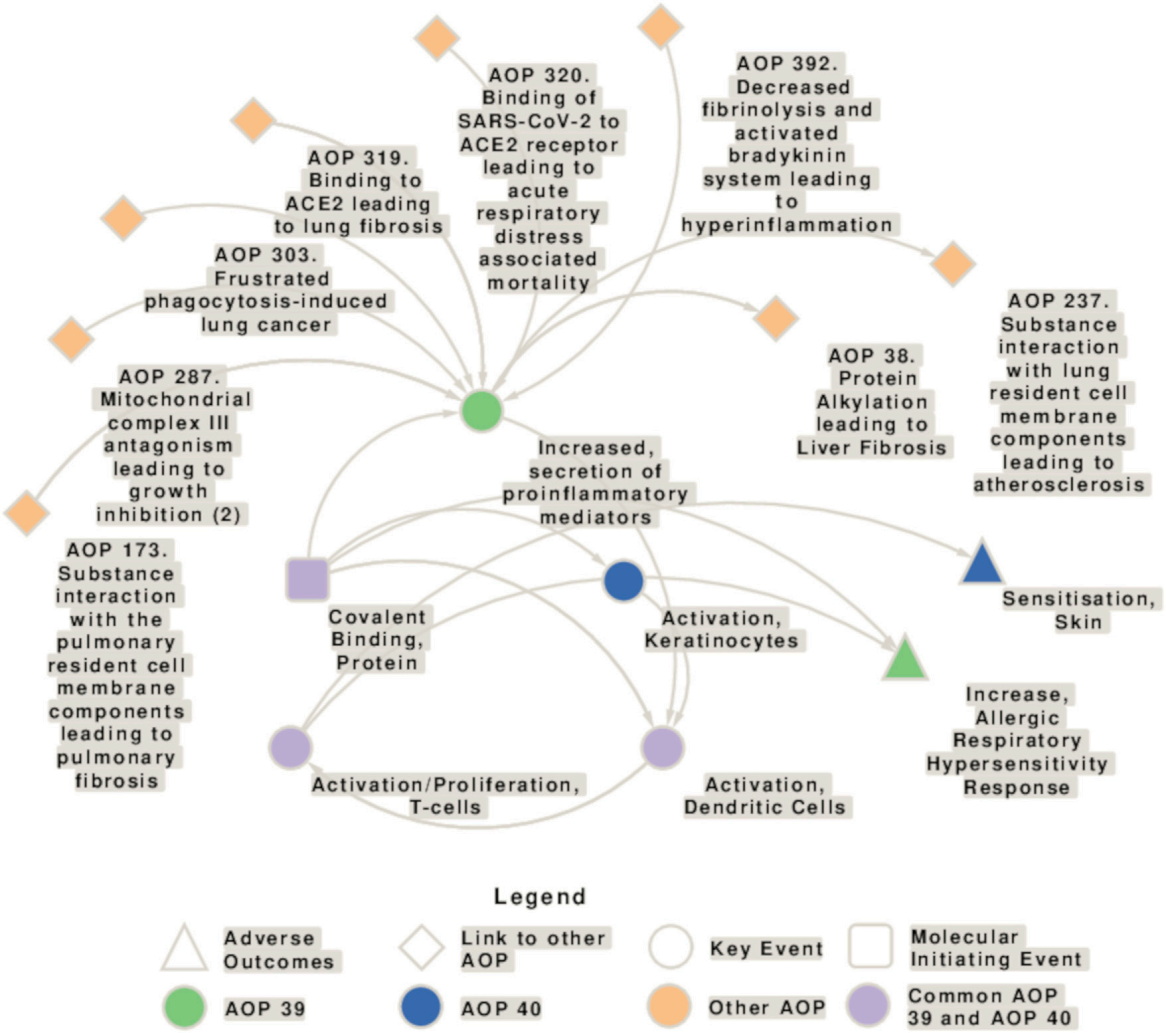
Network showing the interconnection of key events (KEs) involved in the adverse outcome pathways (AOP) for respiratory (AOP 39) and skin sensitization (AOP 40), and connections to other AOPs. AOP 39 and 40 share the molecular initiating event (MIE) “Covalent Binding, Protein,” the key event (KE) “Activation, Dendritic Cells” and the KE “Activation/Proliferation, T-cells.” Moreover, there are some similarities in the AOP 40 KE “activation of keratinocytes” and the AOP 39 KE “Increased secretion of proinflammatory mediators.” KEs specific to AOP 39 are depicted in green, KEs specific to AOP 40 in blue, and KEs present in both AOs in purple. The squares represent the molecular initiating events (MIE)s, circles the KEs, triangles the AOs, and diamonds the connection with other AOPs.

The causal linking of events at different levels of biological organization in adverse outcome pathway (AOP) concepts has evolved as important approach in chemical hazard and risk assessment and is also applied in multiple OECD guidelines. Yet, it is still valid what more than 10 years ago was concluded: although several attempts have been made to understand and assess the effect of respiratory sensitizers in a similar way to skin sensitizers, for which today an AOP (no. 40) and new approach methodologies (NAM) are available (Ezendam et al., 2016), no AOP is established for the identification of respiratory sensitizers and methods need to be developed further, whereby the application of the volatile chemicals and aerosols to the test system is a major issue (Rovida et al., 2013). AOP 39, which is still under development, builds on AOP 40 and addresses a mechanism of action for respiratory sensitizers that is initiated by covalent binding to protein leading to the activation of danger and inflammatory signaling and finally resulting in an allergic respiratory hypersensitivity response (Sullivan et al., 2017; Kimber et al., 2018; AOP-Wiki^2^). The interconnections of key events (KEs) involved in the AOP for respiratory and skin sensitization, and the connections to other AOPs are shown in Figure 1.

AOP 39 provides a summary of the current knowledge regarding the mechanisms underlying respiratory sensitization and aims to identify knowledge gaps that can be addressed by targeted research. In brief, AOP 39 begins with the first key event (KE 1) covalent binding of a LMW chemical to proteins (molecular initiating event, MIE) that leads to the activation of stress response pathways and pro-inflammatory mediators in the form of danger signals, such as oxidative stress, cytokines, and chemokines released by epithelial cells (KE 2), followed by dendritic cell (DC) maturation and migration to lymph nodes (KE 3). Haptens can also directly activate DCs. Migration to the draining lymph nodes of Th2-skewed DCs elicits activation and proliferation of T cells (KE 4), marking the sensitization phase and resulting in chemical respiratory allergy. The increased allergic hypersensitivity reaction is considered the adverse outcome (AO) (Sullivan et al., 2017).

Considerable effort was made to identify respiratory sensitizers in the past, based on patients exposure histories, workplace observations, animal and cell-based studies as well as with *in silico* prediction tools. However, regulatory accepted methods to identify respiratory sensitizers are not available yet (Ponder et al., 2022). Based on structural analysis, the electrophilic nature of sensitizing chemicals is considered to be most relevant for its immunogenicity, whereby specific amino acid residues (cysteine, lysine) are suggested to be the predominant target (Enoch et al., 2009; Enoch et al., 2010). However, different mechanisms may be relevant as well, as, e.g., metals act via complexation (Thierse et al., 2005). Moreover, efforts are made to distinguish between skin and respiratory sensitizers, which is challenging because exposure via skin may also lead to respiratory sensitization and *vice versa* for some chemicals (van Och et al., 2002; De Jong et al., 2009).

We conducted a literature review using artificial intelligence to record to which extent selected molecular events and biological processes, pathological mechanisms, symptoms and adverse reactions are found to be associated with a set of respiratory sensitizers, on the one hand to emphasize the topicality of the issue and, on the other, to obtain information on potential further key events.

Herein, our objective is to elucidate comprehensively the existing knowledge pertaining to respiratory sensitization processes within the context of the AOP framework. Furthermore, we will summarize *in vitro* and *in silico* methods that are potentially applicable for the assessment of respiratory sensitization, though adaptation steps may be required. Additionally, we will identify areas where current understanding is lacking and highlight regulatory concerns that warrant further investigation.

## 2 Methods

### 2.1 Inventory of respiratory sensitizers

Literature-identified respiratory sensitizers (Enoch et al., 2009; Enoch et al., 2010; Rovida et al., 2013; Cochrane et al., 2015; Sadekar et al., 2021; Ponder et al., 2022, ECETOC TR 77^3^) were compared with the official harmonized classification according to Annex VI of the Classification, Labelling and Packaging (CLP) Regulation ((EC) No 1272/2008)^4^ by using the latest consolidated version of Table 3 to Annex VI of CLP^5^ and with the classification of the Classification and Labeling (C&L) Inventory database^6^.

To estimate the overall number of respiratory sensitizers in the C&L inventory, data were download as Excel file on 15 May 2024. To specifically capture data related to respiratory sensitizers, the search query included “health hazards” with classifications “Resp. Sens. 1”, “Resp. Sens. 1A”, and “Resp. Sens. 1B” combined with an “or” operator. The filter was set to include all relevant records without any discrimination. Compounds with no CAS number and records that did not have any classification data, as well all duplicated CAS numbers were removed. Please note that the C&L inventory derived classification categories include harmonized, REACH registration and notified C&L. While the REACH registration C&L is based on self-classification by the registrant(s) and may become an official harmonized classification as soon as a classification dossier is submitted and evaluated, the notified C&L entries were not updated since the submission deadline in 2010 and thus refer to the state of knowledge at that time. In addition, companies and importers may have submitted different proposals indicating inconsistent classification categories for a certain toxicity. The database is continuously updated.

### 2.2 Literature search

To retrieve relevant scientific papers from PubMed, search terms related to respiratory sensitization-relevant biological targets and events that were extracted from AOP 39 and from related AOPs (no. 40 as well as other lung related AOPs), as well as related to adverse outcomes and clinical symptoms were defined. In addition, a small subset of chemical compounds was selected from Supplementary Table 1, and all PubChem listed synonyms for these compounds were used as search term. All search terms can be found in Supplementary Table 2 and are named <query_terms_events>, <query_terms_umbrella> and <query_compounds>. Subsequently, a custom R script, using the “easyPubMed” library (Fantini, 2019), was employed to query for all possible PubMed IDs (PMID) corresponding to each individual compound, event or umbrella term. These queries were constructed in the “[All Fields]” format. To maintain data integrity and eliminate redundancy, any duplicate PMIDs were systematically removed from the retrieved results. Additionally, AOP-helpFinder 2.0^7^ (Jaylet et al., 2023) was used to find relevant publications that connects stressors (the list of compounds) with a merged list of events and umbrella terms. Furthermore, we used the results from AOP-helpFinder to create a stressor-event network. We only included interactions predicted as moderate, high, and very-high, as well as events that occurred more than 5 times. Finally, those publications that were retrieved in both the PubMed and the AOP-helpFinder query were extracted, including information on the publication type (original article or review), and publication year. No further inclusion and exclusion criteria were applied because it is only a status assessment.

### 2.3 AOP 39–40 network

Data from the AOP-Wiki database^2^ release 2.6 was downloaded on 16 June 2023. All KEs belonging to AOP 39 and AOP 40 were retained, and in cases where a KE was connected to another KE from a different AOP, it was relabeled as the associated AOP.

## 3 Identification/classification of respiratory sensitizers and appraisal of the topic in the scientific literature

### 3.1 Comparison of literature-identified sensitizers and official classification

Without prediction methods for the respiratory sensitizing potential of chemicals, it is not possible to estimate the actual extent of the problem, which makes it difficult to take the necessary steps to increase worker and consumer safety. In the past, several initiatives were made to set up reference lists for respiratory sensitizers in order to foster the development of identification methods.

We collected a dataset of 90 compounds identified as respiratory sensitizers from scientific literature (Supplementary File S1) (ECETOC TR 77^3^; Enoch et al., 2009; Enoch et al., 2010; Rovida et al., 2013; Cochrane et al., 2015; Sadekar et al., 2021; Ponder et al., 2022). The assessment of the evidence for the sensitizing properties of the chemicals differs in the literature sources, the respective assessment commentaries can be compared in Table 1. More information about other types of toxicity associated with these chemicals can be found in Supplementary Table 1.

**TABLE 1:** List of respiratory sensitizers identified either by literature search, the official harmonized classification (Annex VI of CLP)^11^ and/or the C&L inventory database^12^.

Chemical	CAS	EC/List no.	Skin Sens. Category (C&L inventory)	Resp. Sens. Category (C&L inventory)	Entry in annex VI of CLP	Ponder et al. (2022)	Sadekar et al. (2021)	Cochrane et al. (2015)	Enoch et al. (2010)	Rovida et al. (2013)	Enoch et al. (2009)	ECETOC TR 77
2-(1H-benzotriazole-1-yl)-1,1,3,3-tetramethyluronium tetrafluoroborate	125700-67-6	423-040-4			No	A						
2-hydroxyethyl methacrylate	868-77-9	212-782-2	1		No		I					
2-methyl-4-isothiazolin-3-one	2682-20-4	220-239-6	1A		No		I					
2,4-Dichloro-5-(chlorosulphonyl)benzoic acid	3740-18-9	223-127-5			No	A						
2,4-Toluene diisocyanate	584-84-9	209-544-5	1	1	Yes				L		S	N
2,6-Toluene diisocyanate	91-08-7	202-039-0	1	1	Yes				L			
4-diazoniobenzenesulfonate	305-80-6	206-168-3			No	A						
5-chloro-2-methyl-4-isothiazolin-3-one	26172-55-4	247-500-7	1		No		I					
6-Amino penicillanic acid	551-16-6	208-993-4			No				L			
7-Amino cephalosporanic acid	957-68-6	213-485-0	1	1	No	A			L			
Abietic acid	514-10-3	208-178-3			No						S	
Ammonium hexachloroplatinate (HCP)	16919-58-7	240-973-0	1	1	Yes	B				P		H
Ammonium Persulfate	7727-54-0	231-786-5	1	1	Yes	A						
Ampicillin	69-53-4	200-709-7	1	1	No	A			L			
Azodicarbonamide	123-77-3	204-650-8		1	Yes		I		L			
Benzyl dimethyl dodecyl ammonium chloride	139-07-1	205-351-5			No		I					
Benzyl n-butyl phthalate	85-68-7	201-622-7			No		I					
C.I. 71105	4424-06-0	224-597-4			No				L			
Carmine	1328-60-5	215-527-3			No	A						
Carminic acid	1260-17-9	215-023-3			No				L			
Cefadroxil	50370-12-2	256-555-6	1	1	No	A						
Cefteram pivoxil	82547-81-7	No results found			No	A						
Cephalexin	15686-71-2	239-773-6	1	1	No				L			
Chloramine T	127-65-1	204-854-7		1	Yes	A	C					
Chlorhexidine	55-56-1	200-238-7			No		U		L			
Chromium	7440-47-3	231-157-5			No			E				
Cobalt	7440-48-4	231-158-0	1	1	Yes			E				
Cyanuric chloride	108-77-0	203-614-9	1		No		I					
Di(2-ethylhexyl) phthalate (DEHP)	117-81-7	204-211-0			No		I					
Dibutyl phthalate	84-74-2	201-557-4			No		I					
Dichlorvos	62-73-7	200-547-7	1		No				L			
Diethyl phthalate	84-66-2	201-550-6			No		I					
Dimethyl ethanolamine	108-01-0	203-542-8			No				L			
Diphenylmethane diisocyanate	101-68-8	202-966-0	1	1	Yes	A	C	E	L	P		N
Epigallocatechin gallate*	989-51-5	619-381-5 and 479-560-7	1		No				L			
Ethanolamine	141-43-5	205-483-3			No				L			
Ethyl acrylate	140-88-5	205-438-8	1		No		Q					
Ethyl cyanoacrylate	7085-85-0	230-391-5			No				L			
Ethylene diamine	107-15-3	203-468-6	1	1	Yes		R	E	L		S	
Ethylene oxide	75-21-8	200-849-9			No		I					
Ethyleneimine	151-56-4	205-793-9			No		I					
Fenthion	55-38-9	200-231-9			No				L			
Formaldehyde	50-00-0	200-001-8	1		No	A	Q				S	
Glutaraldehyde	111-30-8	203-856-5	1A	1	Yes	B	R			P	S	
Glycyl compound	77430-27-4	No results found			No				L			
HBTU*	94790-37-1	619-076-7 and 423-020-5			No	A						
Hexachlorophene	70-30-4	200-733-8			No		I					
Hexahydro phthalic anhydride	85-42-7	201-604-9	1	1	Yes	B	C					
Hexamethylene diisocyanate	822-06-0	212-485-8	1	1	Yes	A	C	E	L	P		O
Himic anhydride	826-62-0	212-557-9	1	1	Yes		R					
Hydroxylamine	7803-49-8	232-259-2	1		No		R					
Isononanoyl oxybenzene sulfonate	123354-92-7	No results found			No		I		L			
Isophorone diamine	2855-13-2	220-666-8	1A		No		I					
Isophorone diisocyanate	4098-71-9	223-861-6	1	1	Yes		I		L			
Maleic anhydride	108-31-6	203-571-6	1A	1	Yes		R		L	P		
Menthol	1490-04-6	216-074-4			No	A						
Methyl cyanoacrylates	137-05-3	205-275-2			No		R		L			
Methyl methacrylate	80-62-6	201-297-1	1		No		I					
Methyldopa	555-30-6	209-089-2			No				L			
Methyltetrahydrophthalic anhydride	11070-44-3	234-290-7	1	1	Yes	B	R					
N-methyl morpholine	109-02-4	203-640-0			No		I					
N-methyl piperazine	109-01-3	203-639-5	1B		No		I					
Naphthalene diisocyanate	3173-72-6	221-641-4	1A	1	Yes		R		L			
Ninhydrin	485-47-2	207-618-1	1B		No		R					
P-phenylenediamine	106-50-3	203-404-7	1		No						S	
Penicillin G	61-33-6	200-506-3	1		No				L			
Persulfate salts (sodium persulfate)	7775-27-1	231-892-1	1	1	No		Q					
Phenylglycine acid chloride	39878-87-0	254-668-5	1	1	No	A						
Phenylglycine chloride	39478-47-2	No results found			No				L			
Phthalic anhydride	85-44-9	201-607-5	1	1	Yes	B	I	E	L		S	N
Piperacillin	61477-96-1	262-811-8	1	1	No	A			L			
Piperazine	110-85-0	203-808-3	1	1	Yes	B	C	E	L		S	
Piperazine dihydrochloride	142-64-3	205-551-2	1	1	Yes		I					
Platinum	7440-06-4	231-116-1			No			E				
Plicatic acid	16462-65-0	No results found			No	B			L			N
Potassium dichromate	7778-50-9	231-906-6	1	1	Yes	A						
Reactive orange 3R	20262-58-2	243-653-9	1	1B	No				L			
Rifazol black GR		No results found			No				L			
Tetrachloroisophthalodinitrile	1897-45-6	217-588-1	1		No				L		S	
Tetrachlorophthalic anhydride	117-08-8	204-171-4	1	1	Yes		R	E	L			
Thiamphenicol	15318-45-3	239-355-3			No	A						
Toluene diisocyanate	26471-62-5	247-722-4	1	1	Yes	B	C	E				
Triethanolamine	102-71-6	203-049-8			No		I					
Triethylene tetramine	112-24-3	203-950-6	1		No		I		L			
Triglycidyl isocyanurate	2451-62-9	219-514-3	1		No		I					
Trimellitic anhydride	552-30-7	209-008-0	1	1	Yes	B	C	E	L	P		N
Tungsten carbide	12070-12-1	235-123-0			No			E				
Tylosin	1401-69-0	215-754-8	1	1	No				L			
Urea formaldehyde	9011-05-6	618-464-3			No		I					
Vinyl benzene	100-42-5	202-851-5			No						S	

Compounds are listed in alphabetical order. References are listed by year of publication, starting with the most recent source. The evidence cited in the sources is abbreviated with letters (A: equal or lower than 10 cases; B: higher than 10 cases; C: compelling evidence; E: occupational asthma linked to clinical and epidemiological evidence; H: no animal evidence, human evidence; I: inadequate evidence; L: clinical studies of individuals with chemical-induced asthma-like symptoms; N: animal and human evidence; P: commented as respiratory positive controls; Q: questionable evidence; R: reasonable evidence; S: respiratory sensitization from frequent literature references; U: uncategorized). The classified respiratory sensitizers listed in the C&L inventory are highlighted in light grey, the ones listed in Annex VI of CLP in dark grey. For comparison, the C&L information for skin sensitization classification is listed, too. Please note that if the cell for the C&L inventory derived classification is empty, this can have different reasons, e.g., (i) no classification due to the lack of data, (ii) some notifiers suggest that the compound has respiratory sensitizing properties, but the majority of notifications does not include this classification, (iii) the CAS number is not available (e.g., for Rifazol black GR), and/or (iv) the compound is not listed in the ECHA C&L inventory database overview (e.g., Cefteram pivoxil). An asterisks label compounds of which the CAS numbers had a double entry in the C&L inventory, the more conservative classification was adopted. This table was updated on 15 of May. 2024.

In order to be as inclusive as possible, we have used all listed chemicals for our analysis including those with low level of confidence or even questionable evidence. This decision was made because individual case reports can still suggest a potential for sensitization, even if they occur with low incidence (Sadekar et al., 2021). This is relevant to extend the AOP network to less frequently described sensitization mechanisms. Each compound was cross-referenced with its CAS number and assessed within Table 3 to Annex VI of CLP and the European Chemicals Agency’s (ECHA) C&L databases for hazard classification. Both the official harmonized C&L, the REACH registration C&L and the notified C&L entries were considered.

The results revealed that out of the 90 literature-derived compounds, 24 compounds have an official harmonized classification according to Annex VI of CLP as respiratory sensitizer (Resp. Sens.) category 1, which indicates respiratory sensitizers for which not sufficient information for sub-categorization is available. According to the C&L inventory, there are additional 8 compounds categorized as respiratory sensitizers category 1 and one compound was classified as category 1B (with a low to moderate potential to cause respiratory sensitization in humans). Furthermore, 19 literature identified compounds were either not classified for respiratory sensitization or not present in the C&L database (see Supplementary Table 1).

Interestingly, the remaining compounds have not received any CLP or C&L respiratory sensitizer classification. Thus, only approximately 27% of sensitizing compounds mentioned in literature are classified as such within the CLP regulation (37% in case of including the C&L inventory derived classified respiratory sensitizers).

**FIGURE 2:**
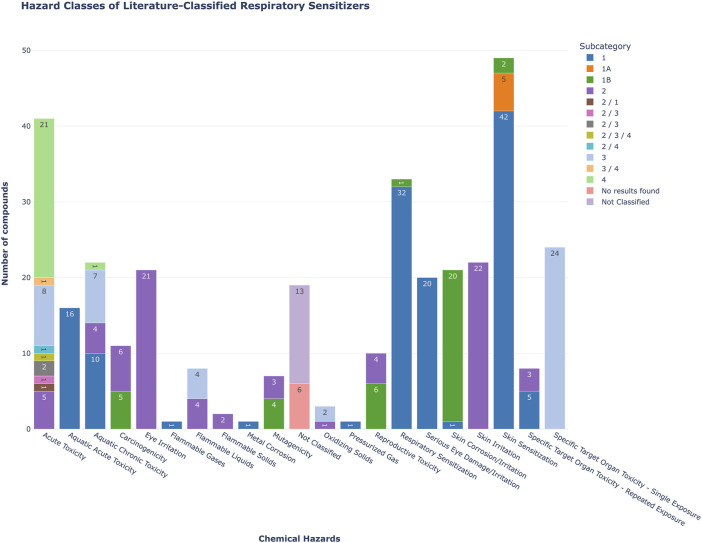
Bar plot showing the hazard classification distribution of 90 compounds previously identified as respiratory sensitizers in the literature, and queried against the ECHA C&L database. The colors indicate the subcategory, the numeric values within the bars indicate the number of compounds associated with specific hazards.

Several of the compounds listed in Table 1 exert also other types of toxicity (Figure 2; Supplementary Table 1). As it is of interest to discriminate between respiratory and skin sensitizers, we compared the compounds in these categories. Among the 90 respiratory sensitizers mentioned in literature, 42 were recorded in the C&L inventory as skin sensitizers category 1, 5 were categorized as skin sensitizers category 1A, and 2 were labelled as skin sensitizers category 1B. When comparing the Annex VI of CLP plus C&L inventory derived classified respiratory sensitizers in our list (*n* = 33), 31 compounds were listed as skin sensitizers, too.

To gain a better understanding of the extent of the respiratory sensitization problem, the number of relevant entries in the C&L database and Table 3 to CLP Annex IV was surveyed. It is important to note that we have not differentiated the type of respiratory sensitizer, thus not only LMW chemicals but all types of sensitizing compounds were included, i.e., protein allergens, particles and mixtures. Overall, currently the C&L inventory contains to date 2296 CAS numbers that are associated with a respiratory sensitization classification. Table 3 to Annex VI of CLP contains 120 entries for respiratory sensitizers.

### 3.2 To what extent is chemical-induced respiratory sensitization reflected in the literature?

In order to better assess the knowledge gained in the last few years around the topic of respiratory sensitization we have adopted a literature search-based approach using PubMed and AOP-helpFinder 2.0, a web tool to identify and extract associations between stressor and event, and between event and event (Jaylet et al., 2023). An overview of the results of the literature search can be found in Figure 4. We have set up query lists containing terms for selected molecular events and biological processes that were mentioned in the literature in the context of respiratory sensitization and insults, and umbrella terms for pathological mechanisms, symptoms and adverse reactions. Moreover, we selected a subset of 6 chemicals (Supplementary File S1), that are both literature-identified and C&L inventory derived classified respiratory sensitizers.

Chloramine T, phthalic anhydride, piperazine, toluene diisocyanate (TDI), trimellitic anhydride and ethylenediamine belong to different electrophilic mechanistic domains (Enoch et al., 2010) and are among the chemicals identified in the “*in litero*” screening as respiratory sensitizers (Sadekar et al., 2021; Ponder et al., 2022). TDI is the prototypical stressor mentioned in AOP 39 due to the availability of information on a concentration-dependent response and temporal concordance with the adverse event. The protein binding mechanism of TDI as well as of phthalic anhydride and trimellitic anhydride is acylation, whereby in particular the anhydrides show a very high electrophilic index (Enoch et al., 2009). Ethylenediamine and piperazine are metabolized to aldehydes, which undergo Schiff base formation (Enoch et al., 2010). N-chlorination is the relevant mechanism for chloramine T (Enoch et al., 2010).

**FIGURE 3:**
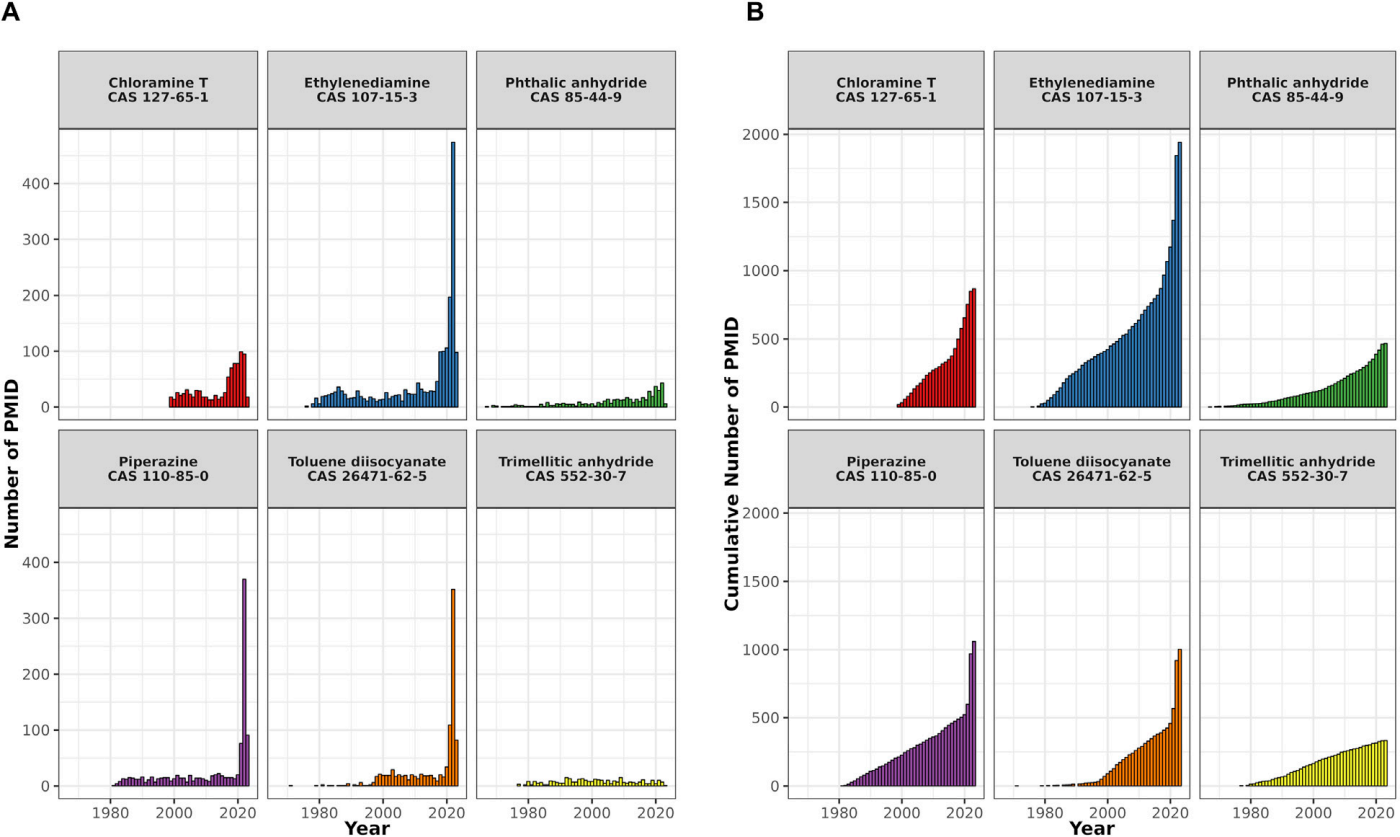
A bar plot showing the temporal distribution of PubMed records (PMIDs) for six selected respiratory sensitizers. The x-axis shows the publication years, the y-axis the number of PMIDs. **(A)** Shows the annual PMID count for each compound, and **(B)** displays their cumulative publication counts.

For all chemicals, PubChem-listed synonyms were included as search terms. The number of publications has shown a consistent upward trend over time for all six compounds, with the most substantial increase occurring in the last 5 years, particularly notable for TDI, piperazine, and ethylenediamine (Figure 3).

**FIGURE 4:**
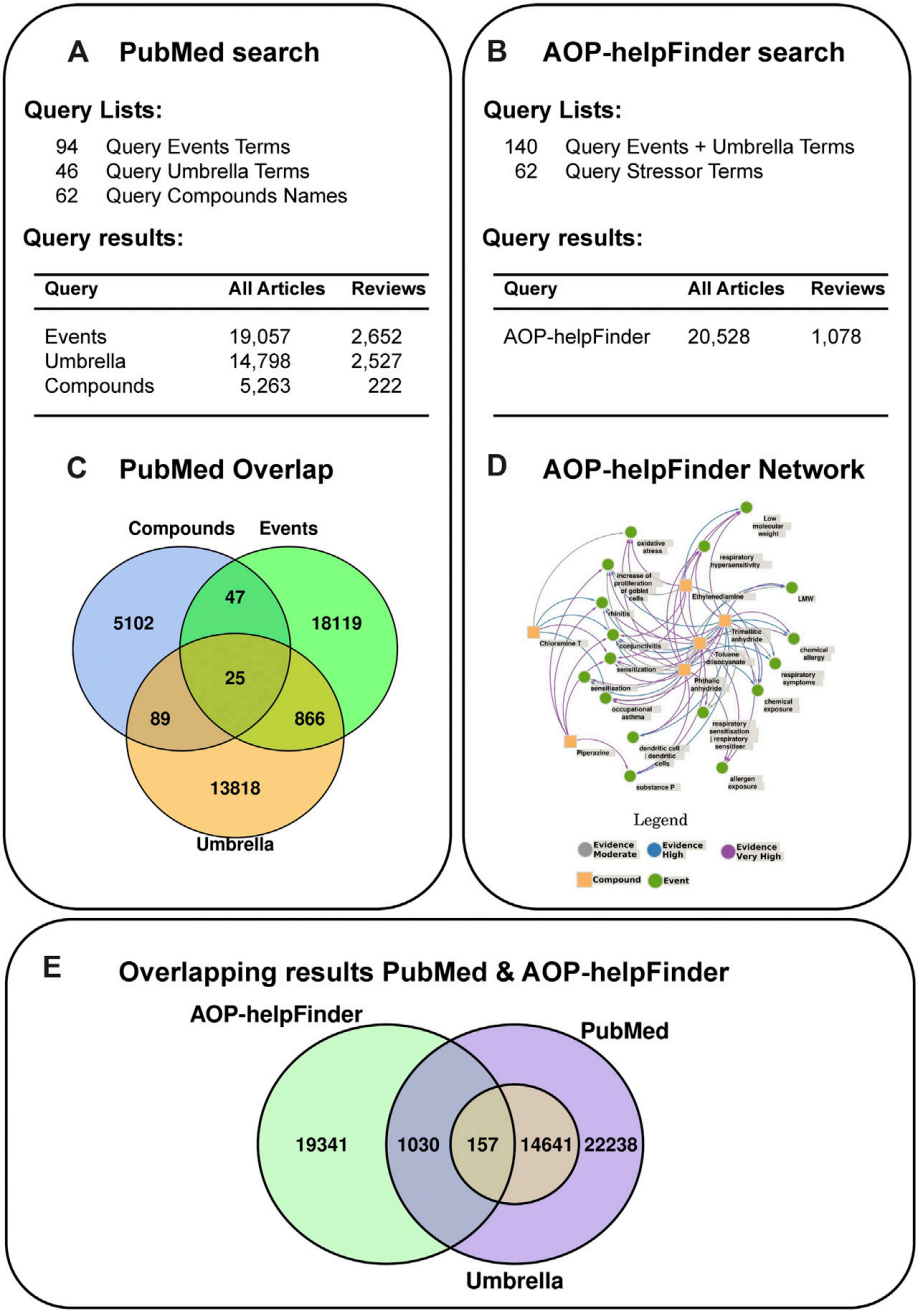
Literature search strategy using PubMed **(A)** and AOP-helpFinder **(B)**. **(A)** The query term lists used for the PubMed search contained 1) events and targets extracted from literature, 2) clinical symptoms and/or adverse outcomes (called umbrella terms) and 3) a number of 6 selected compounds with all synonyms (chloramine T, ethylene diamine, phtalic anhydride, piperazine, toluene diisocyanate and trimellitic anhydride). The number of articles found with the individual query lists are shown. **(B)** For the AOP-helpFinder search, the event and umbrella terms were combined and the list of compounds served as stressors. **(C)** Articles that were found with more than one query list were identified. **(D)** A network was generated based on the predicted interactions strength from AOP-helpFinder. **(E)** A comparison of the results of the two different search strategies revealed 1,187 articles found using both methods.

However, in the PubMed search there is only a minor overlap between the compound associated publications (n = 5263) and the event and umbrella terms of 161 articles (Figure 4C). Yet, the network generated based on the AOP-helpFinder prediction of interaction strength of the compounds with event/umbrella terms (Figure 4D) visualizes the associations with sensitization, oxidative stress and symptoms, i.e., asthma.

## 4 Mechanisms described in AOP 39

As stated above, AOP 39 builds on the skin sensitization AOP 40 and is composed of the KEs: covalent binding of chemicals to proteins (which is the MIE), increased secretion of proinflammatory mediators, dendritic cell activation, activation and proliferation of T cells, finally leading to the adverse outcome (AO) increased allergic hypersensitivity response. This AOP relies on evidence linked to low-molecular-weight chemicals and excluding other known respiratory sensitizers acting via different MIEs (https://aopwiki.org). The prototypical stressor is a low-molecular weight chemical, toluene diisocyanate.

### 4.1 MIE (KE 1): “Covalent binding to protein”, (KE 396)

The MIE represents the first key event (KE 1) in the AOP of respiratory sensitization (Sullivan et al., 2017, AOP-Wiki)^1^. The covalent protein binding - or haptenisation - of respiratory sensitizers was initially confirmed by those respiratory sensitizers, which tested positive in the *in vivo* local lymph node assay (LLNA) for skin sensitizers (Dearman et al., 2013) and subsequently in the *in chemico* Direct Peptide Reactivity Assay (DPRA) test (Lalko et al., 2012), which was developed and validated for the evaluation of KE 1 in the skin sensitization AOP (Gerberick et al., 2004; OECD, 2023a). The LMW chemicals, which induce respiratory sensitization are not able to directly engage with the immune system to elicit an immune response due to their size. Small molecules, haptens, need to bind covalently to a protein and form the hapten-protein complex, which initiates the cascade of events leading to respiratory sensitization. Most respiratory sensitizers are reactive electrophilic compounds or can be converted abiotically by oxidation (pre-haptens) and/or metabolically (pro-haptens) to electrophilic species, which bind to the nucleophilic centers in carrier proteins (Enoch et al., 2009; Enoch et al., 2010). A strong correlation between the presence of multiple reactive functional groups and the ability to induce respiratory allergy has been identified. Respiratory sensitizers were categorized based on their differences in protein binding mechanisms (acylation, Michael addition, Schiff base formation, nucleophilic substitution (SN), however there are also respiratory sensitizers that do not exert an electrophilic mechanism (Enoch et al., 2010).

Different opinions exist regarding the importance of cross-linking properties of chemicals, within or between proteins and the contribution of this mechanism to respiratory sensitization (Enoch et al., 2010; Lalko et al., 2011). Lalko et al. (2012) showed in the DPRA that the majority of respiratory sensitizers favours reactivity with lysine residues, in contrast to skin sensitizers that favour cysteine binding. Lysine is considered the most important biological nucleophile in respiratory sensitization, as cysteine is oxidized in the lung. Moreover, it has been shown that respiratory sensitizers tend to bind to serum albumin, which is rich in lysine residues (Hopkins et al., 2005). Nevertheless, preferential binding of lysine does not represent a prerequisite for respiratory sensitizers, as although the acid anhydrides display this preference (Krutz et al., 2021), other chemical classes do not bind exclusively to lysine (Dik et al., 2016).

Although skin and respiratory sensitizers share several characteristics, such as their electrophilicity, and the chemical reactivity with proteins/peptides in order to be recognized by the immune system, they ultimately lead to different forms of allergic disease. Considering that the adverse effect is differing between the two AOPs, the hypothesis that chemicals form distinct conjugates depending on their specific chemical structure and the nature of the functional groups directs the response of T cells towards preferentially either Th1 or Th2 responses, represents the first point of divergence in the pathways leading to respiratory and skin sensitization, respectively.

Importantly, non-electrophilic compounds and metals do no fall within the applicability domain of AOP 39. Transition metal complexes are non-classical haptens as their sensitization mechanism is based on the formation of coordinated complexes that are not sufficiently strong to survive antigen processing (Chipinda et al., 2011). It is suggested that these complexes either bypass the intracellular antigen processing steps or that protein is used for transport but then short-lived, high-affinity coordination sites are created within certain T cell receptor-major histocompatibility complex (MHC) zones (Thierse et al., 2005). Moreover, some “inert” chemicals are able to bind to the MHC on the basis of their conformation rather than reactivity (Posadas and Pichler, 2007).

The role of lipophilicity and membrane penetration capacity is not widely discussed. Substances that are relatively inert but able to penetrate cell membranes can damage the lung barrier, accumulate intracellularly or are further distributed through the blood system, thereby affecting also other target organs.

### 4.2 KE 2: “Increased secretion of proinflammatory mediators” (KE 1496)

The second KE in AOP 39 refers to activation of inflammatory signaling by substances that promote and regulate inflammation in the respiratory tract. The exposure of the airway epithelium to respiratory sensitizers may cause loss of integrity of the epithelial barriers increasing the permeability of the epithelial cells and gaining access to dendritic cells (DC).

The mechanism of detection of inhaled environmental allergens by airway epithelial cells is realized through pattern recognition receptors (PRRs) which activate different signaling pathways. This leads to production of inflammatory cytokines and chemokines, attracting DCs. The PPRs recognize pathogen-associated molecular patterns (PAMPs) and/or damage-associated molecular patterns (DAMPs). As the lung is continuously exposed to allergens (containing PAMPs and DAMPs), tolerance should occur, however this is not the case in allergic diseases where inflammation is triggered. TLR4 expression on lung epithelial cells, but not on DCs, was necessary and sufficient for the induction of allergic asthma (Hammad et al., 2009). Recognition of PAMPs and DAMPs initiates host defense by triggering the release of the pro-inflammatory cytokines IL-25, IL-33 and thymic stromal lymphopoietin (TSLP), called alarmins. Receptors of these cytokines (IL-17RA, IL1RL1 and TSLPR) are expressed on a wide variety of cells, including DCs and type 2 innate lymphoid cells (ILC2). Activation of DCs and ILCs through alarmins (KE 2) plays key roles in driving type 2 inflammatory responses in asthma by promoting downstream production of type 2 cytokines such as IL-4, IL-5, and IL-13 from the effector cells (KE 3) (Duchesne et al., 2022).

TSLP is known for its role in initiating and maintaining Th2 responses and activating T cells (Soumelis et al., 2002; Ito et al., 2005; Rochman et al., 2007; Akamatsu et al., 2008). Its specific involvement in respiratory sensitization remains uncertain, potentially varying depending on the antigen context (Duchesne et al., 2022). TSLP-activated DCs produce chemokines that attract Th2 cells and stimulate the production of inflammatory Th2 cytokines (Soumelis et al., 2002; Pattarini et al., 2017). TSLP is highly expressed in lung and skin-derived epithelial cells, and certain myeloid DC populations express TSLP receptors (Reche et al., 2001; Ziegler, 2012; Kitajima and Ziegler, 2013; Ochiai et al., 2014). TSLP levels in asthmatic patients correlate with Th2 cytokine expression and inversely with lung function (Ying et al., 2008; Shikotra et al., 2012). OX40L is a Th2 cell-prone costimulatory molecule. Its expression by DCs is used to identify respiratory sensitizers and to discriminate them from skin sensitizers, while for instance the expression of TSLP receptor complex components TSLPR and IL-7Ra did not provide a basis for discrimination between respiratory and skin sensitizers (Mizoguchi et al., 2023). The relevance of OX40L in T cell priming and polarization is discussed in chapter 3.1.4 (KE 4). The Th2 inducing effect of TSLP-matured DCs was further enhanced by another pro-Th2 alarmin, IL-25 (Wang et al., 2007) that is produced by epithelial cells, basophils, and eosinophils (Lambrecht and Hammad, 2009). IL-25, known for its role in parasitic infections, is also linked to asthma and allergy. Various factors such as lipopolysaccharide (LPS), ovalbumin (OVA), and epithelial cell injury trigger IL-25 release. In asthmatic patients, myeloid and plasmacytoid DCs express the IL-25 receptor IL-17RB, which is upregulated after allergen exposure. IL-25 also influences plasmacytoid DC function, impacting TLR9 expression (Tworek et al., 2016).

IL-33, part of the IL-1 cytokine family, is produced by cells in exposed tissues (skin, airways) as well as in lymphoid organs (Mitchell and O'Byrne, 2017). Mice lacking IL-33 show resistance to respiratory allergens (Haenuki et al., 2012; Barlow et al., 2013; Nakanishi et al., 2013). IL-33 activates DCs, promoting Th2 cytokine production by inducing IL-6, IL-1β, TNF, CCL17, and the expression of CD40, CD80, OX40L, and CCR7 *in vitro*. IL-33 recruits and activates DCs in the lungs *in vivo* (Cayrol and Girard, 2022). Another important pro-Th2 and DC attracting cytokine released by epithelial cells during allergic lung inflammation is GM-CSF. This cytokine is crucial for mouse allergic sensitization (Asquith et al., 2008; Su et al., 2008). Overexpressing GM-CSF in airways disrupts inhalation tolerance (Stämpfli et al., 1998). Neutralizing GM-CSF (or IL-33) blocks allergic sensitization to house dust mite (HDM) (Willart et al., 2012). Elevated GM-CSF levels are found in asthma patients (Saha et al., 2009). GM-CSF induces DC maturation (Bleck et al., 2006), impacting Th2/Th17 cell priming and granulocyte recruitment (Nobs et al., 2021). GM-CSF controls resident lung DCs (Greter et al., 2012).

Other cytokines and chemokines such as CCL5, CCL17, CCL11 and CCL22 were also described in different studies to trigger Th2 cell polarization upon exposure to allergens (reviewed by Morianos and Semitekolou, 2020).

Although a multitude of studies demonstrated that all these cytokines were necessary for the development of a Th2 allergic response against an antigen, mounting evidence suggests that the importance of these cytokines in the allergic response varies with the type of the allergen.

Although KE 1496 is not included in AOP 40 (Covalent Protein binding leading to Skin Sensitization; Figure 1), it is reasonable to assume that the increased secretion of pro-inflammatory mediators also occurs in the process of skin sensitization. In the description of the second key event (KE 826) of AOP 40, it is stated that the activated keratinocytes release pro-inflammatory cytokines, such as IL-18, suggesting that similar cellular responses occur after the molecular initiating event.

### 4.3 KE 3: “Activation of dendritic cells” (KE 398)

DCs are professional antigen presenting cells (APCs), characterized by their high surface expression of MHC molecules, co-stimulatory molecules and pattern recognition receptors. They are critical in presenting antigens to T cells, activating them and directing the immune response toward the appropriate immune reaction by migrating to the lymph nodes. DCs in lung are located in virtually all tissue compartments: throughout the conducting airways, in lung parenchyma, alveolar spaces, vasculature, pleura and bronchial lymph nodes (GeurtsvanKessel and Lambrecht, 2008). Immature DCs can exert their guarding role in the lung with direct and indirect sensing of the antigens. DCs can get into direct contact with allergens or haptens at the lung surface even in case the epithelial barrier is not compromised, due to their ability to extend dendrites into the lumen via formation of tight juncions between the epithelial cells lining the alveolar spaces with the aid of claudins and zonula-2 molecules (Lambrecht and Hammad, 2009).

The link between the MIE and activation of DCs in the AOP is well established, but the evidence for the relationship between KE 2 (activation of inflammatory signaling) and DC activation is weak due to a number of data gaps and uncertainties. Lung DCs express receptors for inflammatory mediators and DAMPs released upon tissue damage. In case of respiratory sensitization the primary site of damage is the bronchial epithelial cell layer. Hapten-protein conjugates are taken up by DCs and processed to hapten-modified peptides that can bind to major histocompatibility complex type II (MHCII) molecules (Martin et al., 2010). Antigen uptake and activation of PRRs induces the DC maturation process through which they become efficient T cell stimulators. TLR4 is expressed in abundance on conventional DCs (cDCs,) and monocyte-derived DCs and are important for respiratory sensitization (Trompette et al., 2009; Blecher-Gonen et al., 2019). For example, LPS, a well-known TLR4 ligand, administered as adjuvant in respiratory sensitization experiments using OVA is required to amplify Th2 responses in allergic inflammation (Lamiable et al., 2020). In addition to TLRs, other receptors such as CD301b (Kumamoto et al., 2013), CD206 and dectins (Parsons et al., 2014; Chiffoleau, 2018) were also shown to play an important role in allergen uptake by different subtypes of DCs.

Allergen-bearing DCs are stimulated to migrate to regional lymph nodes (Lambrecht and Hammad, 2010). Mature DCs migrate to the afferent lymph nodes to present their antigens to specific T cells, activate and polarize them towards Th1 or Th2 responses. In case of skin sensitization there are extensive studies available on skin sensitizer-induced DC migration. Fibroblasts mediate migration of cytokine-matured Langerhans cells via different chemokines (Ouwehand et al., 2008; Ouwehand et al., 2012), but for respiratory sensitization this indirect activation of DCs is not completely proven (Sullivan et al., 2017).

It is important to take into consideration that there are different DC subtypes, playing distinct roles in initiating and maintaining allergen-driven Th2 immune responses in the airways. Three well-defined DC populations exist in the lung: two types of conventional DCs (cDCs) and plasmacytoid DCs (pDCs). All three types of DCs are generated from progenitor cells in the bone marrow but all have different functions. Conventional DCs express high CD11c levels and can be divided in two ontogenically different subsets, cDC1s and cDC2s (Musumeci et al., 2019; Wu et al., 2020). cDC1s efficiently promote cytotoxic T cell and Th1 responses, while cDC2 are the most potent in capturing and transporting allergens and inducing Th cell responses (Th1, Th2, and Th17). In addition, cDC2s are important in the allergic response. pDCs participate mostly in the antiviral defense by type I interferon (IFN) production and promote natural killer (NK) cell and T cell responses. pDCs play important roles in regulation of immune tolerance (Lamiable et al., 2020; Xi and Upham, 2020). They are reported to suppress allergic sensitization upon activation by TLR7/TLR9, by promoting the induction of regulatory T cells (Tregs) in mice (Ouabed et al., 2008; Lombardi et al., 2012), thereby regulating respiratory tolerance to inoffensive inhaled antigens (de Heer et al., 2004). The presence of programmed death-ligand 1 (PD-L1) on the surface of pDCs is critical for their suppressive effect. PD-L1-deficient pDCs could not alleviate allergic airway inflammation in mice (Kool et al., 2009).

During inflammation, in addition to the above-mentioned DC subsets, monocyte-derived DCs are recruited from the blood. These inflammatory DCs are very similar to the non-activated CD11b-expressing DCs, but they express the lymphocyte antigen 6 complex C (Ly6C) protein at various levels. CD11b- DCs can directly recognize foreign particles in the airway lumen with their extended processes. In contrast, CD11b+ DCs cannot pass the epithelial barrier, they can only pick up antigens that passed the basal membrane (GeurtsvanKessel and Lambrecht, 2008).

In the steady-state, the sampling and migration of lung DCs to mediastinal lymph nodes does not occur. TLR stimulation is needed to elicit a DC response. Airway epithelial cells might be instructive in causing DC sentinel behavior and activation in the lungs, but it is not known exactly which signals from epithelial cells initiate the sampling behavior of DCs (Hammad and Lambrecht, 2008).

### 4.4 KE 4: “Activation/proliferation of T-cells” (KE 272)

In AOP 39, the evidence is high for the key event relationship (KER) “Activation, dendritic cells leads to activation/proliferation, T-cells” (KER 379; Society for Advancement of AOPs 2023). It is assumed that for an AO to commence, a certain number of DCs is required to be activated and to migrate to the nearest lymph node in order to stimulate the further cascade of biological events. KE 4 of AOP 39 (KE 272) is also included in AOP 40. Its taxonomic applicability is human and mouse, and for both species the evidence is high.

Respiratory sensitization depends on the antigen-driven activation of T cells, being their proliferation and differentiation into effector and memory populations (Martin et al., 2010; Kimber et al., 2018). The mechanisms regarding T cell activation involved in respiratory sensitization caused by LMW chemicals is not fully understood.

In lymph nodes, mature DCs present the processed protein-hapten antigen to the naïve T-cell receptor (TCR) via the MHCII molecule, and if the antigen is recognized as non-self, antigen-responsive T-cells are stimulated to propagate and differentiate into effector T-helper cells (Th1, Th2, Th17) and memory T-cells (Roggen, 2014; Poppleet al., 2016). For the activation and differentiation of naïve T-cells to Th cells TCR-MHCII interaction, as well as interaction between co-stimulatory molecules on DCs and their ligands on T-cells and specific cytokines are needed (Richter et al., 2013; Butcher and Zhu, 2021). The strength of the response depends on the degree of clonal expansion of allergen-responsive T-cells and the number of clones that are able to recognize and respond to the presented antigen (Kimber et al., 2018). If clonal expansion is of sufficient magnitude, sensitization will be acquired.

OX40-L, CD80 and CD86 costimulatory molecules were reported to be necessary in certain steps of Th2 polarization. *In vivo*, OX40L produced by DCs acted as a costimulatory signal for optimal Th2 priming and memory induction, since OX40L-deficient DCs failed to stimulate the expansion and survival of T cells (Jenkins et al., 2007). It was hypothesized that another possible way of activation of DCs towards Th2 responses was reduction of IL-12 secretion (Kimber et al., 2018). It was also reported that *in vitro,* TSLP was able to induce human DCs to express OX40L, but not IL-12. TSLP-induced DC-derived OX40L was required for triggering naive CD4 T cells to produce IL-4, -5, and -13, but in the presence of IL-12, OX40L lost the ability to polarize Th2 cells (Ito et al., 2005). CD80/CD86 co-stimulation on DCs was shown to be only necessary during priming of naive T cells into Th2 cells but not during restimulation of previously primed Th2 cells in the challenge phase (van Rijt et al., 2004).

The cAMP/protein kinase A (cAMP/PKA) signaling pathway is also suggested to be involved in Th2 activation. A decrease of cAMP in mouse DCs resulted in Th2 immune response with an allergic phenotype, whereas increased cAMP induced Th17 immunity (Lee et al., 2015; Kim and Kim, 2018).

Another pathway proposed to be important in Th2 differentiation is the p38 signaling pathway (Hu et al., 2012; Endo et al., 2015). Han et al. concluded that p38 signaling in DCs promoted Th2 differentiation by regulating IL-12 expression (Han et al., 2022).

### 4.5 AO “Increase, allergic respiratory hypersensitivity response” (AO 313)

The adverse outcome “Increase, Allergic Respiratory Hypersensitivity Response” operates at the organ (lung) level, and is the adverse outcome of AOP 39 (AOP-Wiki^2^). The taxonomic applicability of AO 313 includes humans, guinea pigs (*Cavia porcellus*) and rats (*Rattus norvegicus*). High-quality evidence supports its relevance in humans, while for guinea pigs and rats, the evidence is rated as low. AO 313 is relevant during both developmental stages and adulthood, with high-quality supporting evidence (AOP-Wiki).

The most common clinical manifestations of respiratory allergy are allergic rhinitis, rhino-conjunctivitis, sinusitis and asthma. Respiratory allergy symptoms include nasal congestion, cough, sneezing, rhinorrhea, wheezing, chest tightness, shortness of breath, airflow obstruction, and bronchoconstriction (Boverhof et al., 2008; Kuruvilla et al., 2019b; Thá et al., 2021; Xie et al., 2022). Reactions can be acutely life-threatening or lead to chronic occupational asthma.

## 5 Gaps and suggestions for additional KEs and KERs

The AOP 39 already states that there are still unresolved aspects in respiratory sensitization (Sullivan et al., 2017), for some of which research is still needed, but in the meantime some aspects can also be covered by recent knowledge.

### 5.1 Epithelial barrier integrity

Importantly, effects of respiratory sensitizer exposure on lung barrier function are not considered in the current AOP, or only to a limited extent. Yet, exposure effects on barrier integrity including adherens junction proteins and the cytoskeleton have been reported. Moreover, as impaired barrier function forms a hallmark of asthma, this effect may possibly provide a link between respiratory sensitizer exposure and the OA. The role of epithelial barrier function is discussed in more detail in Section 6. The link of impaired barrier function may possibly be extended by the Th2-skewed immune response to respiratory sensitizers in humans and experimental animals, and the intricate relationship between Th2 cytokine production and reduced barrier function. Studies on effects of respiratory sensitizers on barrier function are currently largely limited to TDI, suggesting additional studies should be performed to assess whether this effect is chemical-specific or class-specific. We suggest adding the loss of epithelial barrier integrity as KE. The orphan KE “Loss of barrier function” (KE 1675) in the AOP-Wiki may be filled, accordingly.

### 5.2 DCs

DCs play a crucial role in shaping immune responses to chemical allergens. However, more research is necessary to understand the steps leading to a shift toward Th2 effector responses and sensitization of the respiratory tract and a wider range of chemicals than those currently known needs to be investigated. Moreover, the same DCs can elicit both Th1 and Th2 responses, raising questions about their role in determining the immune response direction and the type of sensitization acquired. It has been suggested that DCs “read” chemical allergens or hapten-protein complexes, tailoring immune responses favoring either Th1 or Th2 types. The reading of the antigen is likely influenced by the nature of the haptenated protein and the characteristics of danger signals and the downstream immunological milieu. Through this mechanism, DCs may trigger responses resulting in sensitization of the respiratory tract (Kimber et al., 2018). In this context, a valuable approach would involve assessing the impact of different DC subsets on sensitization development. This should encompass a comparative analysis of DAMPs, cofactors, and cytokines associated with both skin and respiratory sensitization (Kimber et al., 2018). A comprehensive examination of the transcriptional signature of DCs would shed light on how early changes in DC genes contribute to the expression of maturation markers specific of cDC1 and cDC2.

### 5.3 T cell polarization

Th2 cells are the main drivers of eosinophilic airway inflammation (Kuruvilla et al., 2019b; Arts, 2020). In several experimental studies, chemical respiratory allergens were found to induce a Th2-type immune response (Dearman et al., 2005; Kimber et al., 2018), but other cell types may also contribute to the response (Kimber et al., 2018; Butcher and Zhu, 2021).

Th2 cells release several cytokines, such as IL-4, which stimulates the production of immunoglobulin E (IgE) by B-cells and their clonal expansion and differentiation into plasma and memory B-cells. Th2 cells also release IL-5, which plays a pivotal role in promoting the differentiation and maturation of eosinophil progenitors in the bone marrow, as well as their subsequent mobilization and survival (Menzies-Gow et al., 2007; Rosenberg et al., 2007). Furthermore, they produce IL-9, which enhances mast cell proliferation, and IL-13, which promotes goblet cell metaplasia, increased mucus secretion as well as airway hyperresponsiveness (Chary et al., 2018; Kuruvilla et al., 2019b). The major source of Th2 cytokines is Th2 cells themselves, but other cell types, such as macrophages, basophils, eosinophils and mast cells have also been shown to produce IL-4 and IL-13 upon stimulation (Liang et al., 2011). Secretion of IL-10 by Th2 cells has been suggested to downregulate the DC-derived IL-12 production and thereby leads to Th2 polarization (Aste-Amezaga et al., 1998).

Th17 cells were also shown to play an important role in the immune response through the activation of both contact hypersensitivity and airway hyperresponsiveness characterized by neutrophil inflammation (De Vooght et al., 2013; Roggen, 2014; Xie et al., 2022). IL-17A, IL-21 and IL-22 produced by Th17 cells play a critical role in the non-Th2 type asthma development (Roggen, 2014; Xie et al., 2022). It appears that both human and mouse Th17 cells show a partial Th2 phenotype, expressing IL-4, IL-5, and IL-13, too (Butcher and Zhu, 2021).

Recently, substantial evidence indicates that group 2 innate lymphoid cells (ILC2s) also play a critical role in the type 2 adaptive immune response by producing type 2 cytokines (Gold et al., 2014; Chen et al., 2017; Kuruvilla et al., 2019b). ILC2s are abundant in airway tissues and produce large quantities of IL-5 and IL-13 in response to alarmins released from epithelial cells (Yang et al., 2016; Kuruvilla et al., 2019b). Thus, it has been recognized that innate immunity also has a key role in the pathophysiology of asthma.

We suggest expanding AOP 39 with a KE on T-helper cell polarization and cytokine production. Two similar KEs are already contained in AOP-Wiki, namely, “Increased, activation of T (T) helper (h) type 2 cells” (KE 1499, included in AOP 173) and “Increase of Th2 cells producing IL-4” (KE 1712, included in AOP 314).

### 5.4 B cells and IgE production

The differentiation and clonal expansion of Th2 cells lead to the production of Th2 cytokines that induce immunoglobulin (Ig) class switching to production of antigen-specific IgE by B cells and clonal expansion of naïve and memory B cell populations (Dearman et al., 2003). Immature B-cells could also be directly activated by antigens through the B-cell receptor (BCR) (Dullaers et al., 2012). IgE production can occur both in the germinal centers of regional lymphoid tissues and locally in the airway mucosa, but the extent of germinal center involvement or local IgE production in respiratory sensitization is currently unknown (Chvatchko et al., 1996; Forester and Calabria, 2010; Hoddeson et al., 2010). Some human and animal studies suggest that the respiratory mucosa is the principal site of allergen-specific IgE production during allergic airway inflammation (Dullaers et al., 2012; De Schryver et al., 2015; Nelson and Wu, 2022). Plasma cells produce and release allergen-specific IgE, which has a major role in the elicitation phase. IgE binds to the high affinity IgE receptor (FcεR1), which can be found in the membrane of both basophils and mast cells (Chary et al., 2018; Kuruvilla et al., 2019b). Mast cells reside in the tissues, while basophils are recruited from the circulation to tissues, where they attain final activation (Kuruvilla et al., 2019b).

However, the role of IgE antibodies in chemical respiratory allergy is not as firmly established as in protein respiratory allergy. Many cases of occupational asthma have been reported lacking detectable IgE, which may suggest a possible IgE-independent pathway (Tee et al., 1998; Wisnewski, 2007; Thá et al., 2021). However, lack of IgE could also be caused by the short half-life of serum IgE, or by not having a “spill-over” of the mucosally-produced IgE into the circulation (Dullaers et al., 2012; De Schryver et al., 2015), or it could be due to technical challenges of measuring chemical-hapten specific IgE antibodies (Wisnewski, 2007).

A new KE could focus on B-cell activation, IgE production and binding to high affinity IgE receptors (FcεR1) on mast cells and basophils.

### 5.5 Degranulation of mast cells and basophils

A subsequent single or multiple exposure to the same chemical allergen leads to an accelerated and more vigorous secondary immune response. Memory T-cells are re-activated, producing cytokines and inducing granulocyte infiltration (Thá et al., 2021). Upon re-exposure to the same allergen, antigen can crosslink IgE bound to the FcεR1 on the surface of mast cells and basophils, triggering their degranulation and the release of histamine, lipid mediators (prostaglandin D2, cysteinyl leukotrienes) and pro-inflammatory factors that lead to the clinical symptoms of asthma and rhinitis (Kimber and Dearman, 1997; Fanning and Boyce, 2013).

Nevertheless, the involvement of IgE in chemical respiratory allergy is still controversial, especially in the case of diisocyanates, and it is possible that additional pathways are also involved in the degranulation of mast cells and basophils and the development of chemical respiratory allergy (Selgrade et al., 2012; Kimber et al., 2014; Quirce, 2014).

A new KE might include the evaluation of “Re-exposure to allergens triggers the degranulation of mast cells and basophils”.

### 5.6 Local inflammation and recruitment of inflammatory cells

Local inflammation at the site of exposure is characterized by the influx of lymphocytes and other leukocytes, and the release of inflammatory mediators (Thá et al., 2021). Eosinophils are the principal cell types associated with a type 2 immune response, and large numbers of eosinophils are recruited via the circulation to the site of inflammation following specific pro-inflammatory mediator (cytokine and chemokine) signaling (Bentley et al., 1992; Kimber and Dearman, 1997). Upon stimulation, eosinophils release inflammatory mediators, cytokines, chemokines, granule mediators and cysteinyl leukotrienes (Kuruvilla et al., 2019b). Eosinophils stimulate bronchial fibroblasts to produce extracellular matrix proteins and collagen and thus promote the thickening of the reticular basement membrane (Durrani et al., 2011).

Airway neutrophilia has frequently been reported in patients with isocyanate-induced occupational asthma (OA), as well as in experimental murine models with sensitization through the airways (Matheson et al., 2001; Wilson et al., 2009; De Vooght et al., 2013; Choi et al., 2019; Margelidon-Cozzolino et al., 2022). In contrast, in murine models sensitization through the peritoneum is more likely to prime Th2 response and eosinophilia (Wilson et al., 2009). Neutrophilic asthma is more severe and less responsive to corticosteroids compared with the eosinophilic Th2 type asthma (Choi et al., 2019).

The KE “Local inflammation, influx of lymphocytes and granulocytes” should be included in AOP39. A similar KE is available in AOP-Wiki entitled “Increased, recruitment of inflammatory cells” (KE 1497), which is included in AOPs 173, 303, 377, 392, 409, 451, 468, and 493.

### 5.7 KE related to other AO

**FIGURE 5:**
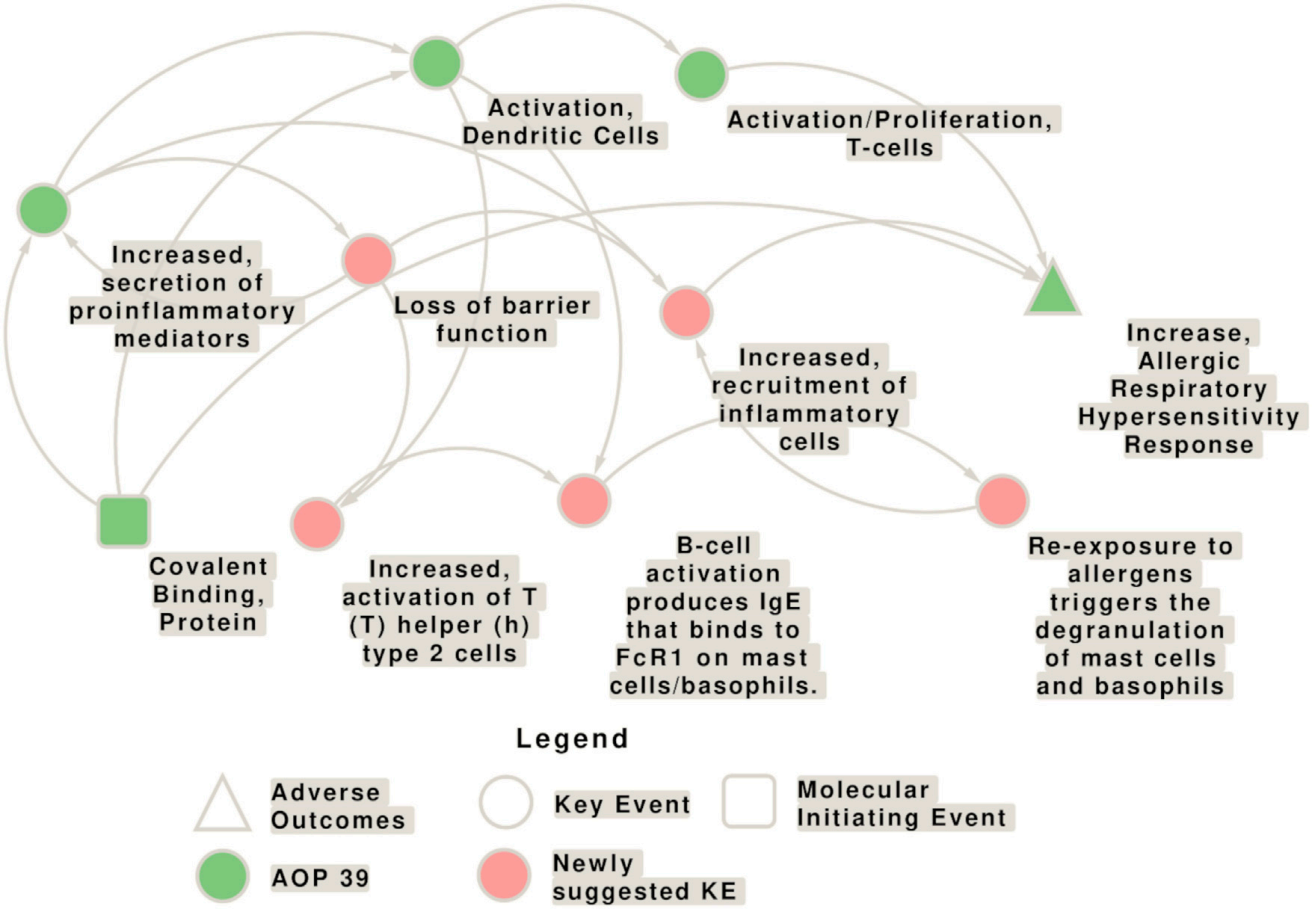
Network of potential interconnections of the key events (KEs) contained in the adverse outcome pathway (AOP) 39 with the newly suggested KEs. The KEs contained in AOP 39 are shown in green, while the newly proposed KEs for this AOP are shown in pink. Yet, the evidence for the relationships, as well as the directionality of the connection needs to be explored in more detail. The molecular initiating events (MIEs) is shown as square, circles represent KEs, and triangles represent the adverse outcomes (AO).

There are several other AOPs which can to a certain extent be related to immune activation in the lung: AOP 196: Volatile Organic Chemicals Activate TRPA1 Receptor to Induce Sensory Pulmonary Irritation; AOP 148: EGFR Activation Leading to Decreased Lung Function; AOP 452: Adverse outcome pathway of PM-induced respiratory toxicity; AOP 272: Deposition of energy leading to lung cancer; AP 411: Oxidative stress Leading to Decreased Lung Function. However, these AOPs have distinct AOs from respiratory sensitization. Moreover, there are several KEs included in other AOPs that could be associated with AOP 39, too, but these KEs need further development (e.g., KE 2010 “Pulmonary inflammation”, KE 2013 “Airway remodeling”, and KE 2086 “Airway inflammation”). The network of potential interconnections of the KEs contained in AOP 39 with the newly suggested KEs is shown in Figure 5.

## 6 The role of the epithelial barrier in respiratory sensitization

Lung epithelium plays an essential role in the protection from environmental insults including pathogens and pollutants/chemicals, as a physical, chemical and immunological barrier. Besides mucociliary escalators and immune defense mechanisms such as secretion of antimicrobial products, the epithelial intercellular junctions are of the utmost importance for the physical separation of the environment from the subepithelial tissue as they regulate paracellular permeability (Ganesan et al., 2013; Hewitt and Lloyd, 2021). Though the cellular composition and epithelial morphology varies depending on the location in the airway, the adhesive forces that maintain physical barrier function are epithelial junctions that consist of adherens junctions (AJs), tight junctions (TJs), and hemidesmosomes (Brune et al., 2015). AJs regulate adhesion of adjacent cells through homotypic interactions between E-cadherin. Disruption of E-cadherin results in delocalisation of TJ proteins. TJs are composed of zona occludens-1 (ZO-1), occludin, claudins, and junction adhesion molecules and are the main regulators of epithelial permeability (Nawijn et al., 2011). A high trans-epithelial electrical resistance (TEER) reflects formation of a tight epithelial barrier, an important aspect of airway epithelial function. TEER measurement can be used to measure epithelial damage due to insults (Gilmour et al., 2023).

Impairment of epithelial barrier function in asthma is a key player in airway inflammation and remodelling (Heijink et al., 2020). Impaired barrier function may allow for higher penetration of allergens, microbes, microbial products, and pollutants across the epithelium, resulting in activation of the immune system and development of allergies (Xiao et al., 2011). Structural changes in the epithelium of asthmatic patients include TJ disruption, and reduced E-cadherin expression. Importantly, the asthma-related cytokine IL-13, produced by Th2 and ILC2 cells, reduced barrier function as well as junctional proteins, including claudin-18, ZO-1, occludin, and E-cadherin (Sweerus et al., 2017; Sugita et al., 2018). IL-13 and the Th2 cytokine IL-4, reduced barrier function and induced physical separation of the TJ molecules, occludin and ZO-1 (Wawrzyniak et al., 2017). Induction of E-cadherin expression reduced the expression of NF-kB, a transcription factor important for airway inflammation (Solanas et al., 2008). Gene knockdown of E-cadherin in bronchial epithelial cells resulted in a pro-inflammatory response, measured as increased expression of TARC and TSLP (Heijink et al., 2007). Airway hyperresponsiveness, another hallmark of asthma, is correlated with airway epithelial damage (Laitinen et al., 1985). Early studies have shown that respiratory sensitizers induce a Th2-skewed response (Dearman et al., 1995; van Och et al., 2002), possibly suggesting effects on epithelial barrier function.

The respiratory sensitizer TDI impaired AJ function, induced E-cadherin redistribution, and increased the permeability of bronchial epithelial cells *in vitro* and *in vivo* (Zhao et al., 2009; Song et al., 2013). Gene profiling of 16HBE human bronchial epithelial cells revealed that exposure to 12 respiratory sensitizers affected expression of genes associated with the cytoskeleton and barrier function (Dik et al., 2015), suggesting that pulmonary barrier integrity is an important target of chemical respiratory sensitizers. Gene profiling of NCI-H292 human pulmonary cells showed that hexamethylene diisocyanate (HDI) exposure resulted in upregulation of thioredoxin reductase, aldo-keto reductase C1, stanniocalcin, and TG-interacting factor (Wisnewski et al., 2002). The first two genes are involved in cellular thiol redox homeostasis. It should be noted that the Wisnewski study may have detected HDI-specific genes and not genes that are representative of a range of respiratory sensitizers.

## 7 New approach methodologies (NAMs) with the potential to be used for the identification of respiratory sensitizers

Due to the urgent need for accurate and reliable test methods for the identification of respiratory sensitizers, various testing methods are being explored. However, there are currently neither *in vivo* nor *in silico* or *in vitro* assays available that are universally accepted and validated. With regard to NAMs, it is proposed that a single test will not be sufficient for a comprehensive assessment, but a combination of suitable assays could be used in an integrated testing strategy (Jowsey et al., 2006; Üzmezoğlu, 2021), similar to skin sensitization^8^. The general requirements for the tests are that they must be standardized, and the definition criteria for positive and negative results must be compared with data from animal studies and/or clinical experience. Most of the *in vitro* and *in vivo* tests described below were developed for skin sensitization, however, their relevance to correctly identify respiratory sensitizers remains to be established.

### 7.1 Computational methods

In recent years, there have been numerous approaches to computer-assisted prediction of the respiratory sensitization potential of chemicals, based on a wide variety of models, some of which are introduced here.

Structure-activity relationship (SAR) analysis is a powerful technique for the prediction of biological properties, including toxicity, of compounds based on their chemical structure. In 2014, Dik et al. evaluated the performance of different SAR models that aimed to predict the respiratory sensitization potential, including those developed by Graham and others using MultiCASE software (Graham et al., 1997), by Cunningham and others using cat-SAR (Cunningham et al., 2005), and by Jarvis et al. (2005) using a logistic regression model. For this, a merged airway dataset was used, which combined data from Derek Nexus (Lhasa Ltd, 2014) and a set of alerts introduced in Enoch et al. (2012) (Dik et al., 2014). The predictivity of the available SAR models for the substances was found to be lower than their published predictive performance, indicating that no single SAR model was sufficiently reliable to draw conclusions about the potential respiratory sensitization properties of a substance. Therefore, it was concluded that the combination with additional computational, *in chimico* or *in vitro* methods is necessary to increase confidence. The profiler developed by Enoch et al. (2014) builds on the understanding of MIEs that lead to organ-level toxicity and aims to predict the respiratory toxicity of LMW chemicals as well as to compare chemical categories. The profiler was developed based on an analysis of 104 chemicals and has been accepted for inclusion in the OECD QSAR Toolbox (Dimitrov et al., 2016).

Golden et al. (2021) evaluated the structural alert model Toxtree, which was originally designed for skin sensitization, and the logistic regression model for occupational asthma from the Centre for Occupational and Environmental Health (COEH). A combined list of recognized respiratory sensitizers (Lalko et al., 2012), a screening-level dataset (Hazardous Substances Data Base; HSDB), and four highly curated chemical respiratory sensitizer datasets (Graham et al., 1997; Jarvis et al., 2005; Enoch et al., 2012; Seed et al., 2015), were included in the analysis. Toxtree had an accuracy of 71% for respiratory sensitization, while the COEH model achieved an accuracy of 76% (Golden et al., 2021).

Recent advancements in machine learning have opened up new possibilities for predicting respiratory sensitizers. Zhang et al. (2018) developed a predictive model for respiratory toxicity using a naive Bayesian classifier based on a dataset of 1241 compounds composed of the Pneumatox database and the dataset from Dik et al. (2014). This dataset was randomly divided between the external dataset (20% of the database) and the training dataset (80% of the database) (Zhang et al., 2018). To ensure the highest prediction accuracy, extended connectivity fingerprints were employed to analyze the structural characteristics of toxic and non-toxic compounds. The model achieved an accuracy of 84.3% when tested on an external dataset (Zhang et al., 2018). Wang et al. (2021) trained six different binary classifiers using various machine learning techniques on a dataset of 2529 chemicals (the Pneumatox database, the Adverse Drug Reaction Classification System (ADReCS) database and the Hazardous Chemicals Information System and compounds mentioned in relevant literature). Among the six techniques employed, support vector machine (SVM) and random forest (RF) performed the best, as indicated by the prediction results of the models.

Voutchkova-Kostal et al. (2022) developed and validated a predictive model for respiratory sensitizers based on mechanistic knowledge within the Computer-Aided Discovery and REdesign (CADRE) platform. They used a dataset of 245 compounds classified as respiratory sensitizers or non-sensitizers from peer-reviewed literature. The model employed a tiered approach, incorporating mechanistic alerts for dermal sensitization and newly developed rules for respiratory sensitizers. In the third tier, a quantum mechanics approach was utilized, considering steric factors and parameters derived from frontier molecular orbitals. The model demonstrated high accuracy, specificity, and sensitivity, with the global quadratic discriminant analysis (QDA) model achieving 93% accuracy and the domain-specific model performing even better at 95% accuracy.

Following the detailed background on computational models for predicting respiratory sensitization, it is crucial to recognize certain limitations in the data sets used for these models. A primary concern is the relatively small number of chemicals conclusively identified as respiratory sensitizers, which poses a challenge in developing robust and accurate prediction models. To compensate, many studies have expanded their data sets to include chemicals with varying degrees of evidence of respiratory sensitization, often encompassing a wider range of respiratory toxicants. This approach, while necessary to increase the size of the dataset for model development, especially in advanced machine learning applications, can lead to ambiguities in the precise categorization of compounds as respiratory sensitizers. Therefore, interpretations of these models must take into account potential limitations arising from both the number and specific labelling of the included compounds.

### 7.2 (Potential) methods for the KEs of AOP 39

Among the laboratory testing methods that have been explored for their potential use in respiratory sensitization assessment (Table 2) is the DPRA, initially designed to determine the protein-binding potential of chemicals in the context of skin sensitization covering KE1 (MIE). The DPRA measures the reactivity of substances towards synthetic peptides containing lysine or cysteine, allowing for their classification into different reactivity classes and identification as sensitizers. The reactivity of a few respiratory sensitizers was tested using this method with positive responses, suggesting that the DPRA can support their identification (Gerberick et al., 2004). As a refinement of the DPRA, the peroxidase peptide reactivity assay (PPRA) has been developed to better discriminate between skin and respiratory sensitizers (Troutman et al., 2011). The PPRA incorporates dose-dependency analyses, mass spectrometry for peptide detection, and a horseradish peroxidase and hydrogen peroxide enzyme system to improve the identification of pro-haptens. However, despite these improvements which allow for better characterization of the reactivity of chemical allergens in general, the data suggest that the PPRA does not provide a significant advantage over the DPRA in distinguishing allergens as skin or respiratory sensitizers (Lalko et al., 2013; Reisinger et al., 2015).

**TABLE 2:** Methods that contain elements that could contribute to address the key events (KE) of AOP 39.

Method name	Test principle	Validation status for skin sensitization	Reference
MIE (KE 1) “Covalent binding, protein”			
Direct Peptide Reactivity Assay (DPRA)	Detection of sensitizers based on their reactivity with synthetic peptides containing lysine or cysteine	OECD TG 442C (2023a)	Gerberick et al. (2004)
Peroxidase Peptide Reactivity Assay (PPRA)	Builds on PPRA using dose-dependence analysis, mass spectroscopy and enzyme system to improve identification of pro-haptens		Troutman et al. (2011)
KE 2 “increased secretion of proinflammatory mediators”			
3D lung models; air-liquid interface cultures	Mimic physiological barrier, can be applied to assess barrier integrity, morphological changes, cytoprotective and cytokine responses, viability, etc.		Lacroix et al., 2018; Sullivan et al., 2017; Thá et al., 2021
KE 3 “activation of DCs”			
Human cell line activation test (h-CLAT)	Measurement of CD86 and CD54 expression by flow cytometry in THP-1 cells	OECD TG 442E (2023b)	Sakaguchi et al. (2006)
Genomic allergen rapid detection test, adaptation for respiratory sensitizers (GARD™air)	GARD Respiratory Prediction Signatures (GRPS) that were established based on the expression of genomic biomarker signatures in MUTZ-3 in a machine learning approach are used to classify test chemicals as respiratory sensitizers	OECD TG 442E (2023b)	Forreryd et al. (2015)
KE 4 “activation/proliferation of T cells”			
Local Lymph Node Assay (LLNA)	*In vivo* assay, measurement of proliferative responses by draining lymph node cells induced following exposure of mice to test chemicals	OECD TG 429 (2010a), OECD TG 442A (2010b), OECD TG 442B (2018)	Dearman et al. (1999)
Human T-cell priming assay (hTCPA)	Measurement of IFN-γ and TNF-α production in cocultures of naïve T cells and chemical-modified/-pulsed monocyte-derived DC after rechallenge		Richter et al. (2013)

The second key event (KE 2) for AOP 39 comprises increased production of cellular danger signals such as inflammatory cytokine, chemokine and cytoprotective gene pathways, and thus shares some similarity with the skin sensitization KE 2 “keratinocyte, activation” as these cells also secrete pro-inflammatory cytokines and induce cytoprotective cellular pathways. For skin sensitizers this can be assessed using keratinocyte activation assays such as KeratinoSens™ and LuSens (Emter et al., 2010; Natsch, 2010; Bauch et al., 2012; Reisinger et al., 2015; Sullivan et al., 2017). Both systems are based on human keratinocyte cell lines carrying a reporter gene construct composed of a luciferase gene under the control of an antioxidant/electrophile response element (ARE), thus addressing the Kelch-like ECH-associated protein 1 (Keap1)/nuclear factor erythroid 2-related factor 2 (Nrf2)/ARE pathway (OECD, 2022). In brief and simplified, an electrophilic modification of the cysteine residues in Keap1 leads to the release of the transcription factor Nrf-2 which shuttles in the nucleus, binds to the ARE containing promoter sequences thereby activating the transcription of cytoprotective genes. For both cell systems, the cell viability is estimated using the 3-(4,5-dimethylthiazol-2-yl)-2,5-diphenyl-2H-tetrazolium bromide (MTT) assay (Bauch et al., 2012; Reisinger et al., 2015). Of note, some respiratory sensitizers were shown to activate the pathway *in vitro*, but both direct induction by attacking of cysteines in Keap1 and indirect activation by changing the redox environment may be relevant (Sullivan et al., 2017). Yet, this pathway is less explored in respiratory sensitization, also due to the preferential lysine targeting of electrophilic respiratory sensitizers. However, the Nrf-2/ARE pathway is important for mediating pulmonary protection against oxidative stress and is involved in the development of lung diseases including asthma (Cho and Kleeberger, 2010).

A number of different lung cell models are available, from simple 2D submerged cultures of different lung epithelial cell lines to 3D models that are suggested to provide more robust, comprehensive responses as they better mimic the *in vivo* situation, in particular when cultivated at the air-liquid interface (ALI) (Lacroix et al., 2018). Such models were used to predict the respiratory toxicity of inhaled drugs based on measurements of viability, barrier integrity, ciliary movement and cytokine release (Mizoguchi et al., 2017; Balogh Sivars et al., 2018). 3D models of the human airway epithelium were also applied to investigate the impact of respiratory sensitizer exposure on cell viability and barrier integrity (Sullivan et al., 2017; Thá et al., 2021). Moreover, sophisticated coculture models composed of lung epithelial cells, macrophages, fibroblasts, dendritic cells are in use, and initial results in respiratory sensitizer analysis are promising (Chary et al., 2019; Mizoguchi et al., 2023).

ALIsens^®^ is a 3D tetraculture model representing the alveolar barrier, which is constructed by a combination of four human cell lines (epithelial cells, macrophages, DCs and endothelial cells) grown at the ALI in hanging cell culture inserts. Following exposure to the test materials, a panel of cell surface markers, chemokines and inflammatory markers and expression of a set of relevant genes are measured allowing the differentiation between respiratory sensitizers and local irritants. The model has been shown to differentiate known sensitizers from irritants and identified correctly the proteins tested so far (Chary et al., 2019).

The ImmuLUNG™ cell model is representative of the alveolar region consisting of alveolar epithelial cells and alveolar-like macrophages (Hutter et al., 2023). The model can be utilized for the detection of irritation and sensitization markers (cell surface markers, cytokines and chemokines). An internal trial conducted with a panel of test items showed promising results for sensitization assessments.

KE 3 requires the investigation of DC activation. Several human myeloid cell lines (e.g., U937, THP-1, MUTZ-3, and KG-1) have been used to gain mechanistic insights and develop predictive assays (Sullivan et al., 2017).

The human cell line activation test (h-CLAT), which is considered one of the most advanced maturation tests for DCs, is included in OECD Guideline 442E (North et al., 2016). Using the THP-1 cell line, the h-CLAT assay measures CD86 and CD54 expression by flow cytometry (Sakaguchi et al., 2006). For skin sensitizers, the h-CLAT showed a good concordance with the local lymph node assay (LLNA), which provides a measure for lymph node proliferation and will be described later as assay relevant for KE 4 (Ashikaga et al., 2010). It was therefore assumed that the h-CLAT is a promising screening tool to assess the potential for respiratory sensitization (North et al., 2016). Recently, a modified h-CLAT assay system was described based on a coculture of THP-1 cells with bronchial epithelial cells that aims to distinguish respiratory from skin sensitizers by measuring expressions of surface markers (CD54, CD86, OX40L) and concentrations of cytokines (IL-8, IL-33 and TSLP) (Tanabe et al., 2023).

GARD*™*air, an adaptation of the genomic allergen rapid detection (GARD) test, provides binary predictions for classifying test chemicals as respiratory sensitizers or non-sensitizers. A human dendritic-like cell line was exposed to reference chemicals, genomic biomarkers were measured and expression signatures were established using pattern recognition and machine learning. Expression patterns of test chemicals are compared with those of the known chemicals. A ring trial evaluating this method demonstrated its high specificity and transferability (Forreryd et al., 2015).

An *in vivo* assay method related to KE 4 (“T-cell activation/proliferation”) is the respiratory LLNA, an adaptation of the validated test method for skin sensitization (Dearman et al., 1999; OECD, 2010). In the respiratory LLNA, mice are exposed by inhalation head/nose-only to the test material during three consecutive days, rather than applying the test material onto the skin of mice. In the respiratory LLNA both contact and respiratory allergens tested positive and could be identified by different cytokine profiles, with the exception of formaldehyde and glutaraldehyde (Arts et al., 2008; Thá et al., 2021).

Although according the current test guideline only proliferation is measured, the LLNA itself would be in fact able to discriminate between skin and respiratory allergens by including an assessment of cytokine levels in the protocol. This is based on the fact that sensitizers induce a divergent immune response, being Th1 vs Th2 for skin and respiratory sensitizers, respectively (Dearman et al., 1995; van Och et al., 2002). Although these models were of some promise, they never gained sufficient traction to support regulatory uptake. Nevertheless, the Th1/Th2 concept for skin vs respiratory sensitizers has been a leading concept since then. To investigate the KE comprising T cell proliferation and activation for contact allergens, the human T-cell priming assay (hTCPA) was used, which measures chemical-specific T-cell frequency and antigen-specific IFN-γ and TNF-α production (Richter et al., 2013). In brief, chemical-modified/-pulsed peripheral monocyte-derived DCs and naïve T-cells derived from peripheral blood of healthy donors are co-cultured in the presence of feeder cells, co-stimulatory CD28 antibody, and cytokines. After 10 days, the secondary response by re-stimulation is measured to ensure that antigen-specific T-cells were primed. Cytokine production by T-cells is detected after a rechallenge with chemical-modified/-pulsed DCs (Richter et al., 2013). This assay is laborious and time-consuming and has high donor-to-donor variability in T-cell repertoire of blood donors, which limits reliability and reproducibility, making the standardization difficult (Martin et al., 2010; Richter et al., 2013; Ezendam et al., 2016; Mizoguchi et al., 2023). A further challenge of this assay is the low frequency of hapten-specific naïve T-cells in the peripheral blood, the potentially high activation threshold of those T-cells, and the delivery of chemicals to avoid toxicity but induce reactivity (Richter et al., 2013). A negative T-cell response may be caused by the failure of the chemical to react with proteins under *in vitro* conditions or because of the lack of T-cells specific for that chemical (Martin et al., 2010). It was found that the removal of regulatory T-cells can significantly improve assay sensitivity, as Treg cells may limit the extent of T-cell responses (Vocanson et al., 2008).

Other measurement methods for KE 4 may include cytokine expression and Th-cell phenotype analysis of draining lymph nodes to determine the proportions of various Th-cell subsets. In addition, cytokine profiling in the bronchoalveolar lavage fluid of lung and in serum would also be informative. Cytokine production by draining lymph node cells excised from chemical-treated mice can be measured after exposure. The preferential type 2 cytokine profile observed after exposure to respiratory sensitizers was associated exclusively with Th2 cell development (Dearman et al., 2003). A protocol variation is to measure IL-4 production following restimulation of lymph node cells with a mitogen (concanavalin A) *in vitro*, but other cytokines (IL-10, IL-13) can also be measured in the absence of re-stimulation (Dearman et al., 2003).

Furthermore, Th-cell phenotype analysis of draining lymph node cells could also be applied to determine the number of each Th-cell subset after exposure to a potential chemical allergen (Zhang et al., 2021).

Mizoguchi et al. developed an IL-4-based 3D co-culture assay consisting of peripheral naïve T-cells and DCs stimulated previously by chemicals in a 3D co-culture system (Mizoguchi et al., 2017; Mizoguchi et al., 2023). The 3D co-culture consisted of an airway epithelial cell line (BEAS-2B), monocyte-derived DCs and a lung fibroblast cell line (MRC-5), and the chemical sensitizer was added on top of the epithelial scaffold (Mizoguchi et al., 2017; Mizoguchi et al., 2023). Shortly after the stimulation, the DC scaffold was removed and placed in a new plate, mimicking the migration of DCs to draining lymph nodes. Allogeneic naïve T-cells were added to the co-culture system and expression of IL-4 was used as a marker of Th2 cell immune response (Mizoguchi et al., 2023). The allogeneic response is generally a polyclonal response and 10% of T-cells are allogeneic T-cells (Mizoguchi et al., 2023). An allogeneic response is stronger than a syngeneic T-cell response, and therefore in this case multiple re-stimulation is not necessary as compared to hTCPA (Mizoguchi et al., 2023). In the DC/T-cell system, respiratory sensitizers, but not skin sensitizers, enhanced expression of IL-4 and the Th2 transcription factor, GATA3 in T-cells (Mizoguchi et al., 2023). The *in vitro* models described by Chary et al. and by Mizoguchi et al. are both promising *in vitro* models to identify respiratory sensitizers, replicating several KEs.

The diagnosis of occupational respiratory allergy in patients as well as the methods used in respiratory epidemiological studies on general population samples are largely based on skin test reactivities (Baldacci et al., 1996) (Table 3). Indeed, atopy is a risk factor for asthma and bronchial hyperresponsiveness. Moreover, several respiratory sensitizers are skin sensitizers as well, which could be an explanation for the usability of the test. However, the extent to which the tests accurately predict allergy against respiratory sensitizers has not yet been systematically recorded.

**TABLE 3:** Methods used in epidemiological research and in clinical practice to address respiratory allergy. Some of these methods or variations thereof are also used with animal models in allergy research and for substance testing.

Method/Abbreviation	Description	Used in animal testing	Reference
Bronchial (methacholine) challenge test/bronchial provocation test (BPT)/nonspecific bronchial responsiveness (NSBR)	Bronchial provocation test to measure bronchoconstriction using spirometry	Shalaby et al. (2010)	Malo et al., 2011, Gupta et al., 2019
Airway hypersensitivity/Specific inhalation challenge test (SIC)	Controlled exposure to a suspected occupational allergen in a dose-progressive manner, as measured by spirometry, pulse oscillometry, and peak expiratory rate		Beach et al., 2007, Gupta et al., 2019
Fractional exhaled nitric oxide test (FeNO)	Measurement of nitric oxide (NO) in exhaled air. NO is produced in airway epithelial cells, eosinophils, and macrophages by nitric oxide synthases. Higher FeNO values indicate eosinophil airway inflammation	Weicker et al. (2001)	Kuruvilla et al. (2019b)
Allergen-specific serum IgE level	Measurement of serum levels of allergen-specific IgE in exposed animals or human patients using immunological assays such as ELISA or the highly sensitive ImmunoCAP platform	Hilton et al. (1996)	Movérare et al., 2017 van Hage et al., 2017
Skin prick test	Intradermal allergen injection, readout is based on mast cell degranulation in the skin by quantifying skin erythema. This test correlates well with serum levels of specific IgE		Baldacci et al., 2001, Gupta et al., 2019
Basophil activation test	*Ex vivo* test that detects allergic reactions by incubating the patient’s blood with suspected allergens and measuring basophil activation, indicated by markers such as CD63 and CD203c, using flow cytometry		Vera-Berrios et al. (2019)
Patch test	An *in vivo* method in which chemicals are applied to the skin and the inflammatory eczematous reaction from delayed hypersensitivity is assessed		Popple et al., 2016 Gupta et al. (2019)

An example is the formation of IgE and also IgG in chemical induced respiratory allergy. For example, Bernstein et al. investigated the ability of trimellitic anhydride (TMA) skin testing to identify sensitized workers and found that skin prick testing was positive in 8 out of 11 workers with serum-specific IgE and intradermal testing in a further two (Bernstein et al., 2011). However, it is not possible to detect allergen-specific IgE in all patients with confirmed chemical respiratory allergy (particularly for diisocyanates) (Tee et al., 1998; Wisnewski, 2007; Thá et al., 2021). This is likely one of the reasons why some chemicals are not classified accordingly (Ponder et al., 2022). Allergen-specific IgE is detectable in 3%–39% of isocyanate-induced asthma patients, the lowest for toluene diisocyanate (TDI) (Tee et al., 1998). In the case of OA, a few weeks away from the workplace or exposure to the chemical may result in decreased serum IgE levels to an extent that it may drop below the detection limit. This situation differs from common environmental aeroallergens (e.g., pollens, dust mite), in which exposure is not restricted to a single place, but allergens are present everywhere at the same time. Therefore, a negative isocyanate-specific IgE assay without accurate exposure information may lead to misdiagnosis (Wisnewski, 2007). Interestingly, IgE can be highly elevated in the airway mucosa independently of IgE serum levels, so analysing local IgE elevation in the absence of systemic IgE could be another approach for diagnosis (De Schryver et al., 2015).

Other *ex vivo* cellular/biochemical methods for the detection of an allergic hypersensitivity response (not described in AOP 39) include (1) the measurement of total IgE in serum with ELISA (Baldacci et al., 2001; Rodriguez del Rio et al., 2022; Xie et al., 2022), (2) absolute eosinophil count (AEC) measured in whole blood, nasal secretion and sputum (Baldacci et al., 2001; Kuruvilla et al., 2019b; Rodriguez del Rio et al., 2022), (3) periostin level measured in serum as a biomarker of persistent eosinophilic airway inflammation (Kuruvilla et al., 2019b; Rodriguez del Rio et al., 2022), and (4) the level of various eosinophil-derived cationic cytotoxic proteins (eosinophil peroxidase (EPO), eosinophil cationic protein (ECP), eosinophil-derived neurotoxin (EDN) in serum, saliva, bronchoalveolar lavage fluid (BALF), and nasal secretion (Baldacci et al., 2001; Rodriguez del Rio et al., 2022). As an *ex vivo* cell-based assay, the lymphocyte transformation test (LTT) measured in PBMCs or T-cells isolated from blood, could indicate the presence of allergen-reactive memory T-cells (Popple et al., 2016). Moreover, transcriptomics from the bronchial tissue, sputum and blood (Kuruvilla et al., 2019b), and metabolomics measured in exhaled air (Kuruvilla et al., 2019b) could also provide a promising biomarker(s) of asthma.

Diagnosing OA can be challenging, therefore, innovative tests and a combination of multiple readouts are required to improve diagnostic accuracy (Tarlo et al., 2008). Some of the *in vivo* methods mentioned in Table 3 or variations of them have been applied in animal models, too, to assess outcomes relevant for respiratory allergy. Even though the main focus is to avoid animal experimentation as far as possible, these methods need to be discussed, since on the one hand animal experiments can also be carried out in reduced settings, and on the other hand the pathophysiological rationale behind the test methods could provide indications for the establishment of new *in vitro* methods.

The assessment of infiltrating cells, cytokines and chemokines in sputum and BALF may provide further mechanistic information. For example, recruitment of pro-inflammatory cells can be measured using BALF cellularity (Siddiqui et al., 2008; Yu and Chen, 2018) and deliver quantitative information on the types of infiltrating cells, and cytokine and chemokine levels. Eosinophil count in induced sputum was shown to increase after exposure during the Specific Inhalation Challenge (SIC) test in which patients are exposed to a suspected occupational allergen in a dose-progressive manner in a controlled setting (Üzmezoğlu, 2021). The SIC test is widely used and considered the “gold standard” in the diagnosis of OA (Tee et al., 1998; Beach et al., 2007; Gupta et al., 2019; Pemberton and Kimber, 2021; Üzmezoğlu, 2021). In the case of TDI, some studies reported increased neutrophil count in induced sputum, but the predictive value of the changes in the neutrophil count has not been established yet (Park et al., 1999).

### 7.3 Methods to assess the newly suggested KE

Methods for the assessment of the newly suggested KEs are available, or could be developed based on tests used currently for research purposes.

Measuring trans-epithelial electrical resistance (TEER) is a method for quantifying the integrity of tight junctions in cell culture models of epithelial and endothelial monolayers (Srinivasan et al., 2015). A high TEER reflects the formation of a tight epithelial barrier, an important aspect of airway epithelial function and can be used to measure epithelial damage due to insults.

For the suggested KE on T-cell polarization and cytokine production the hTCPA could be applied. Another model could be the IL-4 based 3D co-culture assay, which measures Th2 response following chemical stimulation in a co-culture system with airway epithelial cells, monocyte-derived DCs, lung fibroblast cells and naive T cells (Mizoguchi et al. 2023).

The suggested KE on B cells and IgE production could be addressed by measuring allergen-specific serum IgE levels in exposed animals, for which a variety of immunological assays such as ELISA or the highly sensitive ImmunoCAP platform are available (Movérare et al., 2017; van Hage et al., 2017).

Information on the degranulation of mast cells and basophils may be derived from skin prick tests (Baldacci et al., 2001; Gupta et al., 2019) as well as from basophil activation tests, an *ex vivo* test that detects allergic reactions by incubating the patient’s blood with suspected allergens and measuring basophil activation, indicated by markers such as CD63 and CD203c, measured using flow cytometry (Vera-Berrios et al., 2019).

The KE on local inflammation and recruitment of inflammatory cells can be addressed by animal experimentation with the patch test (Popple et al., 2016; Gupta et al., 2019), as well as assessment of infiltrating cells, levels and types of cytokines and chemokines in the BALF (Dearman et al., 2003; Siddiqui et al., 2008; Yu and Chen, 2018; Kim et al., 2019) and by using lung histopathology with, e.g., precision cut lung slices (PCLS) that allow the assessment of local inflammation and recruitment of inflammatory cells following exposure to chemicals (Siddiqui et al., 2008; Kim et al., 2019).

## 8 Conclusion

Respiratory sensitization is a highly topical issue with an urgent need for action, especially in the regulatory field due to its relevance as immune status that triggers occupational disease. In order to achieve this, it is necessary to identify and classify respiratory sensitizing chemicals with the best possible certainty, however this requires a thorough understanding of the underlying chemical and molecular mechanisms, and the existence of valid test systems.

For immune system-mediated skin sensitization and skin irritation there is a clear separation in classification, though skin sensitizers may also induce skin irritations at higher percentages. Different *in vivo* and *in vitro* validated test protocols are available. This is not the case for immune system-mediated respiratory sensitization and for respiratory irritants because of the lack of validated tests. For the CLP classification there is no need to differentiate between these two groups as ‘an immunological mechanism does not need to be demonstrated’.

Protecting workers and the general population is paramount, warranting precautionary measures even for compounds with unknown mechanisms of action. This approach may lead to an over-classification for safety’s sake, prioritizing worker protection over strict mode of action-based classification. This can be understood as a commitment to safety despite incomplete mechanistic understanding, advocating for further investigation into compounds identified as potential risks.

Thus, there are dual aspects at play: regulatory considerations and the necessity for enhanced mechanistic understanding to foster improved protection measures. Ultimately, the aim should be to advance the understanding of mechanisms to refine both classification and regulation.

For this, understanding the physicochemical properties of respiratory sensitizers is essential. In addition to lipophilicity, which determines where most of a substance is deposited in the respiratory tract, electrophilicity is an important property of many, but not all, chemicals that are suspected of having a sensitizing effect on the respiratory tract. Electrophilicity is relevant for the formation of immunogenic haptens, but is not a sufficient distinguishing feature from skin sensitizers. In addition to classical hapten-forming electrophiles, there are non-classical haptens like transition metal complexes that do not fall within the applicability domain of AOP 39. The immune-mediated mechanism of “inert” chemicals that are able to bind to the MHC on basis of their conformation rather than reactivity has also not yet been well explored. Moreover, it is not known to what extent the potential of a substance to penetrate the epithelial layer can contribute to the sensitization process, as compounds can have an indirect effect on immunological reactions by affecting cellular signalling cascades and metabolism.

One major uncertainty related to the respiratory sensitization pathway is the route of exposure through which sensitization of the respiratory tract can be achieved. As mentioned in the introduction, it was demonstrated that beside inhalation exposure, also skin exposure to relevant chemical allergens can effectively sensitize the respiratory tract, supported by animal studies and clinical cases (Tsui et al., 2020). Thus, the type of sensitization and resulting allergic reactions induced by chemical allergens do not solely depend on the exposure route.

Consequently, theoretically proposed predictive test methods for identifying chemical respiratory sensitizers would not need to be limited to inhalation exposure or interaction with respiratory tract cells. It needs to be investigated if methods based on skin or even epithelial exposure in general could be valid for identifying chemical respiratory allergens. The use of skin reactive methods to identify respiratory allergies in clinical practice would support this conclusion.

Some KE are common for both respiratory and skin sensitization, which may explain that typical respiratory sensitizers such as diisocyanates and acid anhydrides also tested positive in skin sensitization tests. Otherwise, certain contact allergens do not typically lead to respiratory sensitization, and *vice versa*. Some chemical respiratory allergens are rarely or never linked to skin sensitization. The question is to which extent such characteristics can be modelled by NAMs. To answer this, a comparison of the performance of known skin and respiratory sensitizers in the different methods addressing different KEs would be required.

For example, in an ALI model the respiratory sensitizers OPA, TMA, and HDI all induced OX-40L expression, differentiating them from skin sensitizers, but also showed sensitizer-specific upregulation of ST2 (OPA) *vs* TSLPR and IL-7R (TMA and HDI) (Mizoguchi et al., 2023). Conversely, some chemicals can trigger both types of reactions. As stated in 7.2, the inclusion of cytokine measurements could enable a better discrimination, which is currently not taken into account in the guidelines, as the LLNA, for example, is used exclusively for proliferation.

To distinguish between contact and respiratory allergens, it is crucial to investigate potential chemical distinctions. Conducting a thorough comparison of the structural attributes of contact and respiratory sensitizers is essential for this purpose. Different classes of chemicals will generate different types of haptens, probably inducing also distinct mechanisms. Moreover, the direct penetration of the epithelial membrane by compounds and their potential to induce intracellular changes of the redox environment may be relevant for the activation of immunological and danger signals in a hapten-independent manner. Among the herein newly suggested KEs for AOP 39 is the assessment of barrier integrity as reduced barrier function is a hallmark of asthma, and has been convincingly shown for some respiratory sensitizers such as TDI. However, whether this is a common effect of respiratory sensitizer exposure awaits further study.

The next question that needs to be answered is whether the methods mentioned have the potential to cover the newly suggested KEs. As many of these methods currently require animal experiments, innovative approaches will need to be developed that further reduce or replace animal testing. Furthermore, although AOP 39 states that there is high evidence for the taxonomic applicability in mouse and human, this cannot be extrapolated to the new KEs without proof, as there are considerable differences in the immune system of the lung. The question is whether sufficient mechanistic understanding is already available to cover key mechanisms with *in vitro* or *in silico* methods. A particular challenge is that for immunological responses, intercellular communication is of great importance, whereby not only different immune cells interact but also the surrounding tissue. Further research is needed to better understand the basic aspects of intercellular communication in the response to respiratory sensitizers.

A major constraint in the development of further methods is the uncertainty concerning the immunological mechanisms. For example, there is no consensus whether respiratory hypersensitivity depends on IgE-mediated mechanisms, since in some cases of chemical-induced asthma (e.g., diisocyanates) only a minority of patients display detectable IgE (Tee et al., 1998; Wisnewski, 2007). Moreover, the identification of respiratory sensitization as cause of disease in clinical practice is a major challenge. Less frequently occurring clinical symptoms are currently not taken into account in the regulatory classification of respiratory sensitizers. This could also be one of the reasons why there is only a partial overlap in our comparison of the respiratory sensitizers mentioned in the literature with the current official classification. Thus, there may be more substances that do not meet all criteria to be classified according current legislation or for which data is lacking, or for which there is inconsistent data, or the classification process is still ongoing. Moreover, there is no measure for the potency of a respiratory sensitizer, as exposure is often based on statistical coincidence only. It is unknown to what extent individual susceptibility plays a role. This not only points to the difficulty of identifying the substances themselves, but also makes it difficult to estimate the real problem size of chemical respiratory sensitization.

Knowledge of the effects of exposure to respiratory sensitizers on human health is still limited, both in terms of occupational exposure and, to an even greater extent exposure in daily life. It is even more difficult to determine causalities, especially in the case of mixed toxicities. For instance, chemical and building-related intolerance are associated with chemical exposure and inflammatory airway disorders including asthma (Anderson and Anderson, 1999; Claeson et al., 2018), as well as multiple chemical sensitivity (MCS), an adverse multisystem response to common chemicals at low doses considered non-toxic for the general population. Due to the difficulty in diagnosis such disorders are often approached from the psychiatry-psychosomatic side and not from toxicology (Hill et al., 2010; Rossi and Pitidis, 2018; Hempel et al., 2023). MCS patients may represent a group of individuals being particularly vulnerable to exposures. The role of respiratory sensitizers in the etiology of such diseases remains to be investigated.

The factors that affect the susceptibility to development and/or the clinical presentation of respiratory hypersensitivity are largely unknown but it can be assumed that factors relevant for asthma in general are important, such as genetic polymorphisms, the individual immune status and microbiome, respiratory virus infections, age (immunoscenesce), air pollution and smoking, presence of indoor allergens, co-medication e.g., betablockers that may cause bronchoconstriction, and co-morbidities such as obesity (Kuruvilla et al., 2019b). Sex differences need to be considered too, as, e.g., it is known for severe asthma that there is a shift from male to female predominance after adolescence (Zein and Erzurum, 2015). In addition, psychosocial factors such as stress can affect lung development, neuroendocrine, autonomic and immune responses and thus increase reactivity to allergens (Rosenberg et al., 2014).

To summarize, we suggest to integrate the newly suggested KEs in AOP 39 to get a more complete picture of the impact of test chemicals on the different steps involved in respiratory sensitization. Yet, this AOP alone may not be sufficient for all respiratory sensitizers as it is limited by the MIE to electrophilic chemicals that form haptens, excluding, e.g., transition metals. In addition, a strategy is needed to differentiate between lung and skin sensitizers. Thus, further research is needed to decipher the relevant mechanisms for respiratory sensitization in more detail. The concentration-dependency of responses needs to be considered as for some chemicals lower concentrations lead to sensitization while at higher concentrations irritation prevails. Thus, also the exposure time and metabolic properties of the cell systems is of importance. Finally, the awareness of respiratory sensitization as a cause for disorders also needs to increase. Further translational and clinical research is needed as diagnosis of respiratory sensitizer-triggered diseases such as OA is still a major challenge.

## Footnotes

1
https://www.oecd.org/chemicalsafety/testing/oecdguidelinesforthetestingofchemicals.htm

2
https://aopwiki.org

3
https://www.ecetoc.org/wp-content/uploads/2014/08/ECETOC-TR-077.pdf

4 Classification, Labelling and Packaging (CLP) Regulation ((EC) No 1272/2008): EUR-Lex - 02008R1272-20231201 - EN - EUR-Lex (europa.eu).

5 Annex VI to CLP_ATP20: https://echa.europa.eu/information-on-chemicals/annex-vi-to-clp

6
https://echa.europa.eu/information-on-chemicals/cl-inventory-database

7
https://aop-helpfinder.u-paris-sciences.fr/

8
https://www.ecetoc.org/wp-content/uploads/2014/08/ECETOC-TR-077.pdf.
